# General anesthetic action profile on the human prefrontal cortex cells through comprehensive single-cell RNA-seq analysis

**DOI:** 10.1016/j.isci.2023.106534

**Published:** 2023-03-31

**Authors:** Enqiang Chang, Yangyang Wang, Ruilou Zhu, Lingzhi Wu, Yitian Yang, Shuang Zeng, Ningtao Li, Xiaoguo Ruan, Mingyang Sun, Wei Zhang, Jun Zhou, Mengrong Miao, Hui Zhi, Hailin Zhao, Qian Chen, Qizhe Sun, Emer Chang, Albert Chang, Tingting Zhang, Xinfang He, Kan Liu, Songhua Ma, Weizhong Zhu, Youming Zhang, Luca Magnani, Daqing Ma, Jiaqiang Zhang

**Affiliations:** 1 Department of Anaesthesiology and Perioperative Medicine, Center for Clinical Single Cell Biomedicine, Henan Provincial People’s Hospital, People’s Hospital of Zhengzhou University, Zhengzhou, Henan, China; 2 Division of Anesthetics, Pain Medicine and Intensive Care, Department of Surgery and Cancer, Faculty of Medicine, Imperial College London, Chelsea and Westminster Hospital, UK; 3 Center for Clinical Single Cell Biomedicine, Henan Provincial People’s Hospital, People’s Hospital of Zhengzhou University, Zhengzhou, Henan, China; 4 Department of Gynecology and Obstetrics, Henan Provincial People’s Hospital, Zhengzhou, Henan, China; 5 Department of Physiology, School of Medicine, Nantong University, Nantong, Jiangsu, China; 6 Section of Genomic and Environmental Medicine, National Heart and Lung Institute, Faculty of Medicine, Imperial College London, London, UK; 7 Division of Cancer, Department of Surgery and Cancer, Faculty of Medicine, Imperial College London, London UK; 8 National Clinical Research Center for Child Health, Zhejiang, China

**Keywords:** Neuroscience, Techniques in neuroscience, Transcriptomics

## Abstract

The cellular and molecular actions of general anesthetics to induce anesthesia state and also cellular signaling changes for subsequent potential “long term” effects remain largely elusive. General anesthetics were reported to act on voltage-gated ion channels and ligand-gated ion channels. Here we used single-cell RNA-sequencing complemented with whole-cell patch clamp and calcium transient techniques to examine the gene transcriptome and ion channels profiling of sevoflurane and propofol, both commonly used clinically, on the human fetal prefrontal cortex (PFC) mixed cell cultures. Both propofol and sevoflurane at clinically relevant dose/concentration promoted “microgliosis” but only sevoflurane decreased microglia transcriptional similarity. Propofol and sevoflurane each extensively but transiently (<2 h) altered transcriptome profiling across microglia, excitatory neurons, interneurons, astrocytes and oligodendrocyte progenitor cells. Utilizing scRNA-seq as a robust and high-through put tool, our work may provide a comprehensive blueprint for future mechanistic studies of general anesthetics in clinically relevant settings.

## Highlights

• Gene transcriptomes in human brain cells treated with anesthetics
• Transcriptional changes of ion channels after anesthetics exposure
• Anesthetics-induced ion channels and calcium transient changes
• Providing blueprint for future mechanistic study of anesthetics

## Graphical abstract

**Figure:**
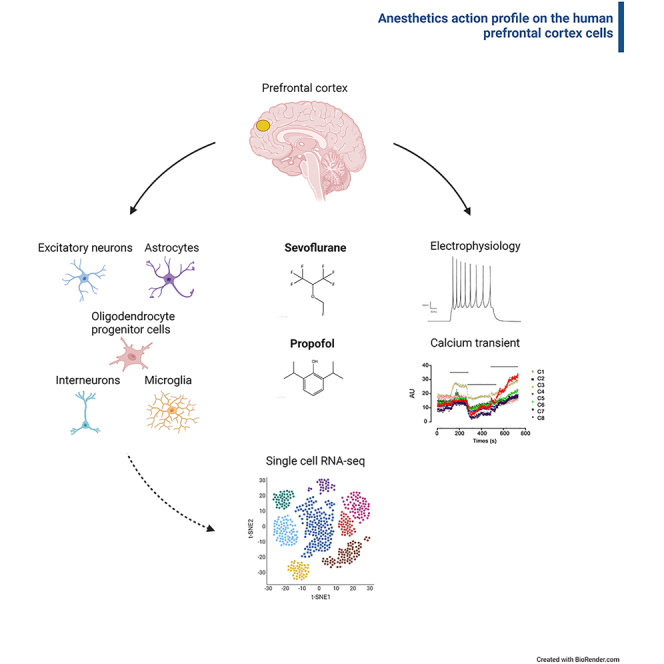

## Introduction

General anesthetics have been used to induce anesthesia state during surgery for more than 150 years. Research being conducted in this field has built the foundation for developing more effective and safer compounds that elicit rapid induction and recovery from anesthesia. Their cellular and molecular modes of action have been investigated primarily through *in vitro*, *in vivo* and *ex vivo* animal models and their potential long-term effects in those settings on the central nervous system have recently been recognised.^1^^,^^2^^,^^3^^,^^4^^,^^5^^,^^6^

General anesthetics have the rapid onset of action owing to their high lipid solubility and imminent effects on cell membrane ion channel conductance, which are currently considered to be the main target of anesthetics.^3^^,^^4^^,^^7^^,^^8^^,^^9^ Indeed, general anesthetics were reported to act on voltage-gated Na^+^, K^+^ and Ca^2+^ channels, ligand-gated ion channels such as γ-aminobutyric acid (GABA) receptor and N-methyl-D-aspartic acid (NMDA) receptor.^1^^,^^2^^,^^3^^,^^4^ A recent study showed that action potential discharge of glutamatergic neurons of the retrotrapezoid nucleus is strongly enhanced by inhalational anesthetic isoflurane. The ionic mechanism includes the activation of an unidentified Na^+^-dependent inward current as well as the inhibition of a background K^+^ current.^10^ Similar findings were made with sevoflurane in mice.^3^^,^^10^ However, these studies on neuronal membrane proteins or related secondary messengers in rodents have not provided convincing evidence to explain the pharmacodynamic characteristics of general anesthetics in humans.^11^

In this study, we cultured brain cells derived from the human prefrontal cortex (PFC), which governs the highest-order cognitive functions including memory, decision-making capability, and social behavior,^12^^,^^13^^,^^14^^,^^15^^,^^16^^,^^17^^,^^18^ of deceased human fetuses, as only brain cells at such developmental stage can be cultured under current technology for experimental utilization. PFC cells were then exposed to a clinically relevant concentration of inhalational anesthetic sevoflurane or intravenous anesthetic propofol followed by single-cell RNA sequencing (scRNA-seq), whole-cell patch clamp, and calcium transient analysis. Our study provides a comprehensive blueprint for understanding the molecular, transcriptome, and functional effects of general anesthetics on the different cell types within the human prefrontal cortex, and facilitates future studies to establish the mechanistic basis of the functional sequelae of general anesthetics in humans.

## Results

### Brain cell populations affected by sevoflurane and propofol

**Figure 1:**
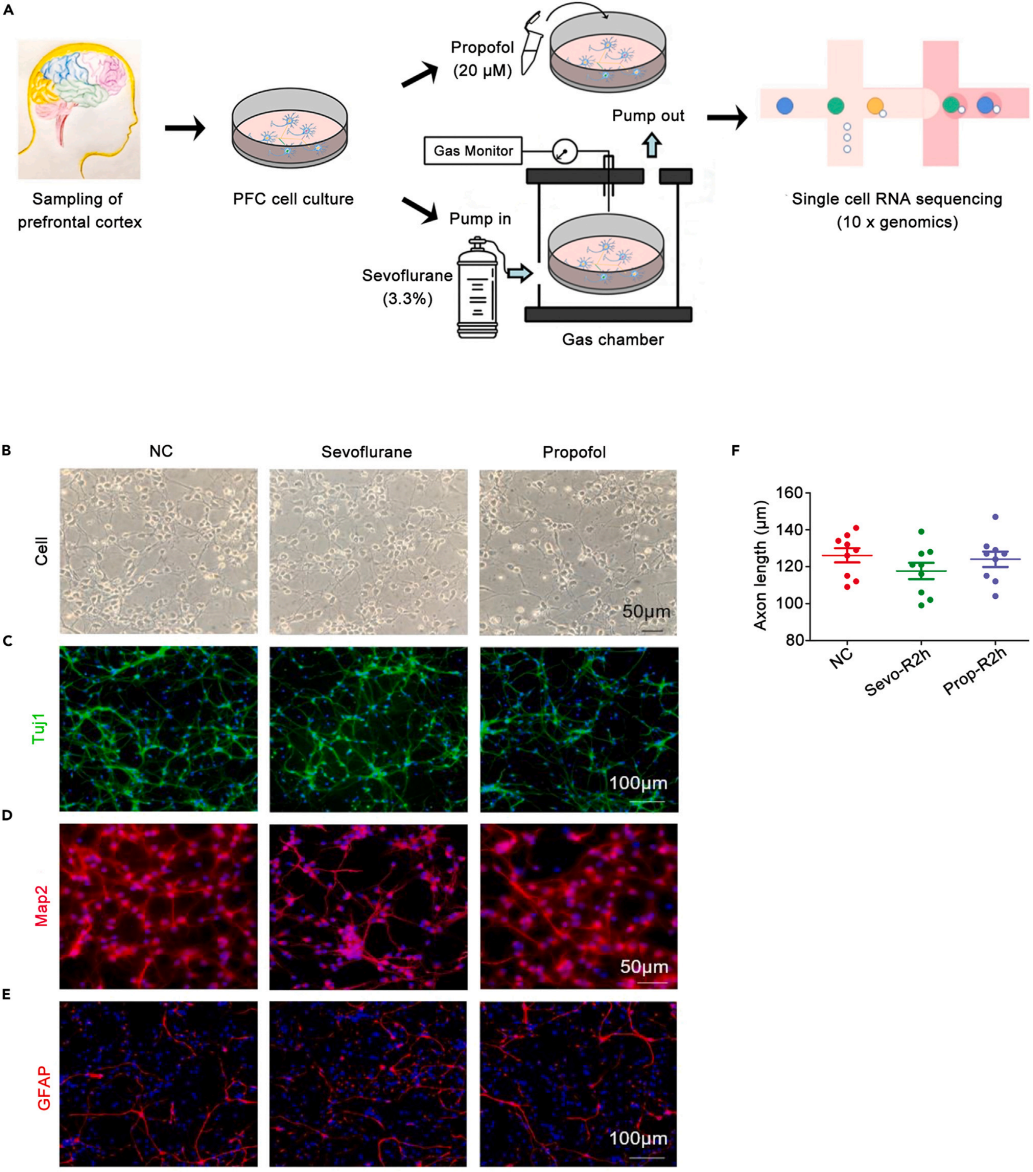
Experimental workflow and prefrontal cortex cells of human brains cultured *in vitro* (A) Experimental workflow of single-cell RNA-seq of human developing PFC by sevoflurane and propofol treatment. (B) Prefrontal cortical cells viewed under light-field microscope, from left to right: NC, Sevo 6h, and Prop 6h treated group. Scale bar, 50 μm. (C) Immunofluorescence staining showing Tuj1-positive PFC cells in NC, Sevo 6h, and Prop 6h groups. Scale bar, 100 μm. (D) Immunofluorescence staining showing Map2-positive PFC cells in NC, Sevo 6h, and Prop 6h groups. Scale bar, 50 μm. (E) Immunofluorescence staining showing GFAP-positive PFC cells in NC, Sevo 6h, and Prop 6h groups. Scale bar, 100 μm. (F) Compared with NC, the length of axons did not change significantly by anesthetic sevoflurane or propofol (p > 0.05).

**Figure 2:**
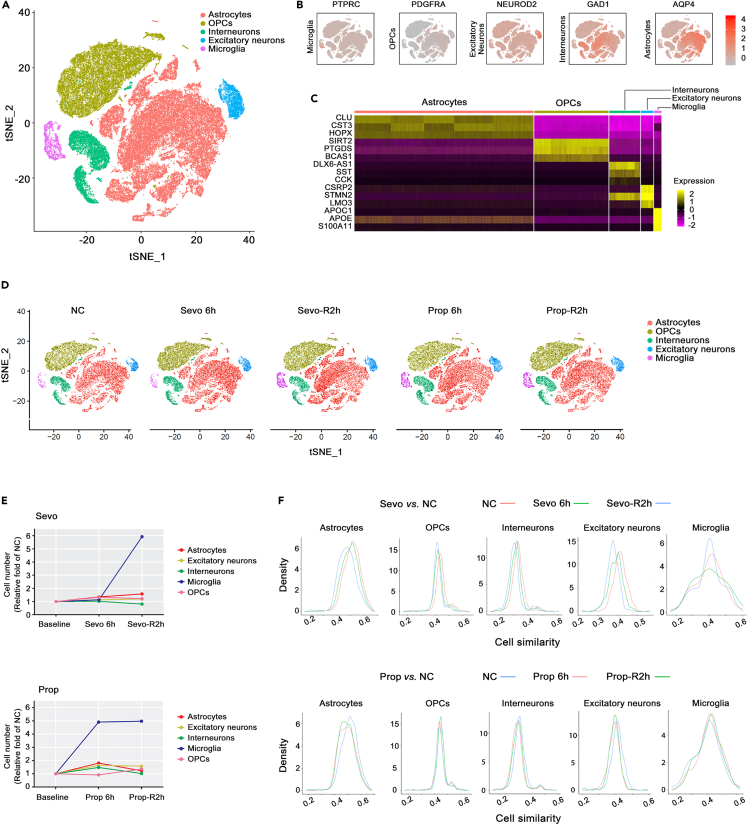
Prefrontal cortical cell population and molecular signature affected by sevoflurane and propofol Single-cell RNA-sequencing, cell number counting, and similarity analyses were carried out after anesthetic exposure in cultured human brain cells derived from prefrontal cortex. (A) Visualization of major classes of human PFC cells using *t-SNE*, clustered by differential marker gene expression. Each dot represents an individual cell. n = 11,600-14800 cells each group. PFC, prefrontal cortex. tSNE: t-distributed stochastic neighbor embedding; OPCs: oligodendrocyte progenitor cells. (B) Relative expression of known marker genes: gray (low expression), red (high expression). The expression patterns were consistent with the annotations of each cell subgroups: PTPRC for microglia, NEUROND2 for excitatory neurons, GAD1 for interneurons, AQP4 for astrocytes, and PDGFRA for OPCs. *PTPRC*: Protein Tyrosine Phosphatase Receptor Type C; *PDGFRA*: Platelet-Derived Growth Factor Receptor Alpha; *NEUROD2*: Neuronal Differentiation 2; *GAD1*: Glutamate Decarboxylase 1; *AQP4*: Aquaporin 4. Red, upregulation; gray, downregulation. (C) Heatmap of top three expressed genes for different types of primary brain cells. Genes for astrocytes: CLU, CST3 and HOPX; genes of OPCs: SIRT2, PTGDS and BCAS1. genes for interneurons: DLX6-AS1, SST and CCK; genes for excitatory neurons: CSRP2, STMN2 and LMO3; and genes for microglia: APOC1, APOE and S100A11. Yellow, upregulation; purple, downregulation. (D) Visualization of different groups of PFC cells using *t-*SNE and the number of different cell types. Dots, individual cells; color, cell types. n = 11,600-14800 cells/group. (E) Fold changes of cell clusters at different time points. After 6 h exposure to sevoflurane, the number of microglia did not change significantly, but after 2 h of recovery, microglia population increased by 4 times from the baseline (NC). Immediately after 6 h treatment with propofol, the proportion of microglia increased by nearly 4 times, without further changes in number after 2 h of recovery. (F) Similarity of cell clusters at different time points following sevoflurane or propofol treatment. Amongst all cell types, microglia population exhibited the most obvious deviation from NC after sevoflurane treatment. Propofol did not alter microglial cell similarity, however cell similarity for astrocytes and excitatory neurons was different from that of NC.

In this study, the brain cells derived from the prefrontal cortex of deceased fetuses at 28 weeks gestation were cultured, and cell-type specific markers (PTPRC for microglia NEUROND2 for excitatory neurons, GAD1 for interneurons, AQP4 for astrocytes and PDGFRA for oligodendrocyte progenitor cells) were applied^19^ to distinguish these cells into five distinct groups by scRNA-seq analyses (Figures 1A, 1B, 2A, and 2B). Of the 22,153 genes analyzed by scRNA-seq, the top 3 expressed genes based on copy numbers from each cell cluster were mapped and showed no overlap, except STMN2 being highly expressed by excitatory neuron and interneuron clusters. Therefore, the cell-type markers selected sufficiently differentiated the different cell populations in a mixed culture (Figure 2C).

*In situ* immunostaining showed that these cells were positive for Tuj1, Map2 or GFAP (Figures 1C-1E). Exposure to sevoflurane or propofol for 6 h, which is the upper “threshold” of routine anesthetic exposure for surgeries, did not change the length of axons of neurons (p > 0.05) when compared to controls (Figure 1F). ScRNA-seq also revealed that the PTPRC-positive population expanded following sevoflurane or propofol exposure, implying the increased number of microglial cells. A significant increase in microglia population occurred for 6 h propofol exposure and sustained throughout 2 h recovery, but this increase induced by sevoflurane was only remarkable during 2 h recovery relative to the control cultures with mock treatment at each corresponding time point (Figures 2D and 2E). Moreover, the cell transcriptional similarity analysis within each cell cluster revealed that, unlike propofol, sevoflurane decreased microglia transcriptional similarity (Figure 2F).

### Gene and enrichment pathways affected by sevoflurane and propofol

**Figure 3:**
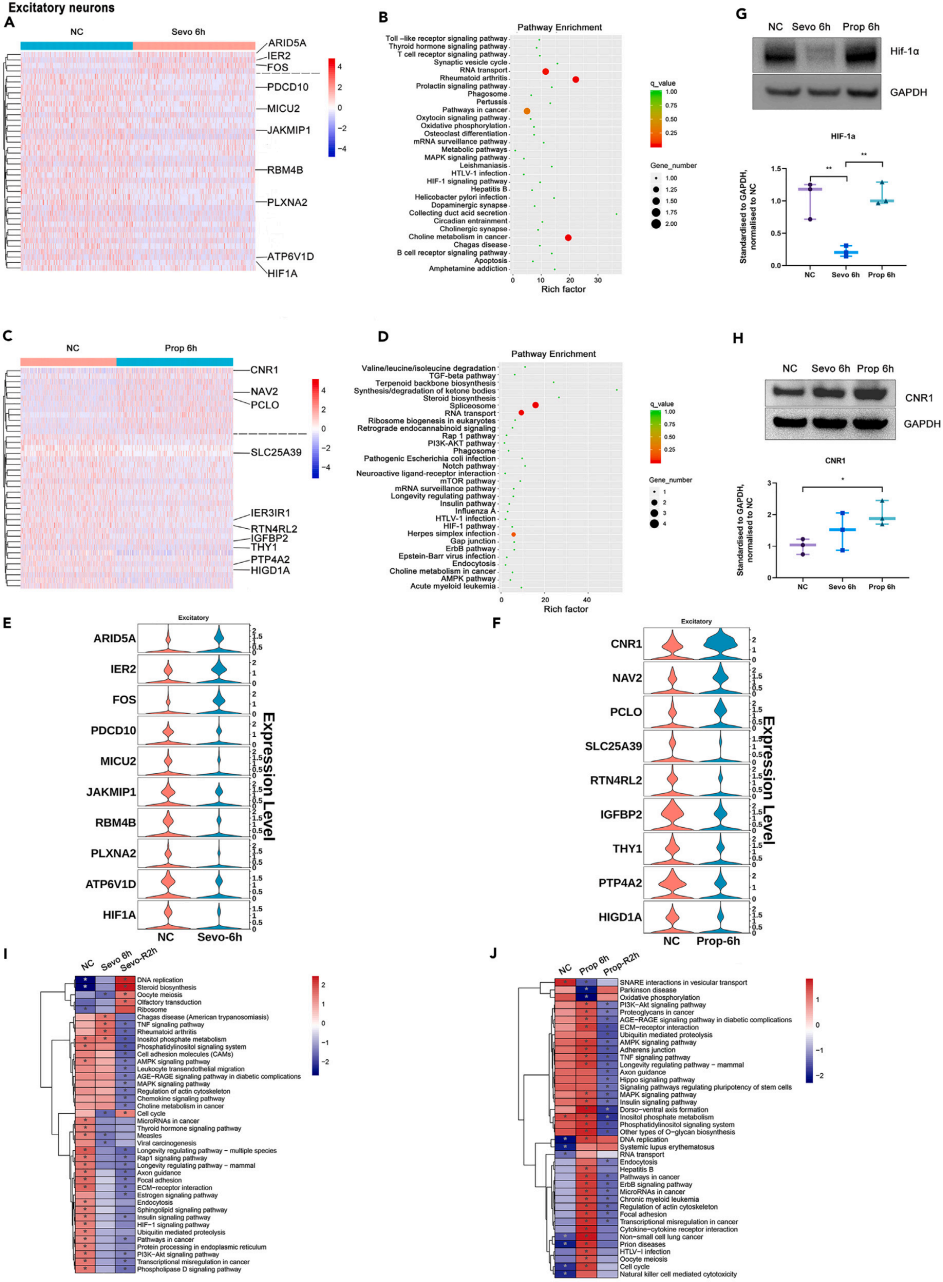
Differentially expressed genes (DEGs) and translational change analysis, enriched pathways analysis, and Gene Set Enrichment Analysis (GSEA) of excitatory neurons (A) Heatmap showing differentially expressed genes in excitatory neurons from Naive control (NC) and after 6h of sevoflurane treatment (Sevo 6h). Red, upregulation; blue, downregulation. (B) Enriched pathways by DEGs analysis in excitatory neurons from NC and 6 h of sevoflurane treatment. (C) Heatmap showing differentially expressed genes in excitatory neurons from the NC and after 6h of propofol treatment (Prop 6h). Red, upregulation; blue, downregulation. (D) Enriched pathways by DEGs analysis in excitatory neurons from NC and 6 h of propofol treatment. (E) Violin plot showing the top 10 differentially expressed genes in excitatory neurons from Naive control (NC) and after 6h of sevoflurane treatment (Sevo 6h). (F) Violin plot showing the top 10 differentially expressed genes in excitatory neurons from the NC and after 6h of propofol treatment (Prop 6h). (G) Western blot analysis of HIF-1α expressions on neurons extracts. ∗∗p < 0.01. Student T-test was used. (H) Western blot analysis of CNR1 expressions on neurons extracts. ∗p < 0.05. Student T-test was used. (I) GSEA of excitatory neurons from NC, Sevo 6h, and sevoflurane treatment for 6 h and recovery for 2 h (Sevo-R2h). Red, upregulation; blue, downregulation. ∗p < 0.05. (J) GSEA of excitatory neurons from NC, Prop 6h and propofol treatment for 6 h and recovery for 2 h (Prop-R2h). Red, upregulation; blue, downregulation. ∗p < 0.05.

The top 40 regulated genes and top 30 enriched pathways by gene ontology analysis during sevoflurane or propofol exposure and following recovery in five types of PFC cells are presented herein (Figures 3A-3D and S1-S5). In excitatory neurons, sevoflurane strongly upregulated *ARID5A* (AT-Rich Interaction Domain 5A, an eukaryote conserved transcriptional factor involved in cell growth and tissue-specific gene expression) and the classical neuronal immediate-early genes *IER2* (Immediate-Early Response 2) and *FOS* (Fos Proto-Oncogene). A handful of genes suppressed by sevoflurane included *MICU2* (Mitochondrial Calcium Uptake 2, mitochondrial calcium uniport regulator), *JAKMIP1* (Janus Kinase And Microtubule Interacting Protein 1, microtubule-dependent transport of the GABA-B receptor), *RBM4B* (RNA Binding Motif Protein 4B, translational activator of circadian clock mRNA *PER1*), *ATP6V1D* (ATPase H+ transporting V1 subunit D, component of vascular ATPase for the acidification of intracellular organelles) and *HIF1A* (hypoxia-inducible factor 1 subunit alpha, a transcriptional factor that orchestrates metabolic adaptation to hypoxia (Figure 3E and Table S1). Propofol exposure induced a distinct set of genes from sevoflurane, include *CNR1* (cannabinoid receptor 1, mediating the mood and cognition alteration effects of cannabinoids. *NAV2* (neuron navigator 2, cellular growth and migration), *PCLO* (piccolo presynaptic cytomatrix protein, component of presynaptic cytoskeletal matrix to enable synaptic vesicle trafficking). On the other hand, propofol potently downregulated *SLC25A39* (Solute Carrier Family 25 Member 39, inner mitochondrial membrane transporter), *IER3IP1* (Immediate-Early Response 3 Interacting Protein 1, endoplasmic reticulum stress sensor that mediates cell differentiation and apoptosis), *RTN4RL2* (Reticulon 4 Receptor-like 2, cell surface receptor inhibiting axon outgrowth), and *THY1* (Thy-1 cell surface antigen, immunoglobulin involved in cell adhesion/communication of the nervous system) (Figure 3F and Table S1). In order to validate the transcriptional changes at the translational level, the protein expression of *HIF1A* and *CNR1* induced by anesthetics were selected for further determination. The immunoblotting data showed that HIF-1α (Figure 3G) was downregulated by sevoflurane whilst CNR1 expression was upregulated by propofol (Figure 3H). These were corroborated with the transcriptome finding reported above. Interestingly, after 2 h recovery, there is a tendency for the altered transcriptome to return to baseline, for example *ARID5A, IER2, FOS.* and *CNR1* (Figures S1A and S1B). There was enrichment for pathways responsible for spliceosome, RNA transport, and neuroactive ligand-receptor interaction (Figures 3B and 3E). After 2 h of recovery, there was also differential enrichment in pathways associated with morphine addiction, axon guidance, and steroid biosynthesis (Figures S1A and S1B).

The heatmaps generated from gene set enrichment analysis (GSEA) showed that sevoflurane and propofol differentially regulated gene expression across 5 different cell types. Sevoflurane had mixed effects (up/downregulation) on gene expression of excitatory neurons during 6 h exposure, i.E. genes associated with cell cycle and Axon guidance were inhibited and TNF signaling pathway were activated. During 2 h recovery post-sevoflurane exposure the majority of gene expression declined to below baseline levels such that they were downregulated relative to naive controls (Figure 3I). In contrast, propofol appeared to upregulate gene expression of almost all enriched pathways in excitatory neurons during 6 h exposure, preceding a systemic downregulation during 2 h recovery (Figure 3J). Upregulated pathways included PI3K-Akt, ECM-receptor interaction, AMPK, TNF, Dorsoventral axis formation, and phosphatidylinositol and focal adhesion appeared to be activated during 6 h of propofol exposure. Only SNARE interaction in vesicular transport, Parkinson’s disease-related genes, and oxidative phosphorylation were downregulated. The transcriptome changes in other brain cell types including astrocytes, interneurons, microglia, and oligodendrocyte progenitor cells (OPCs) exposed to sevoflurane and propofol were comparable (Figure S6).

**Figure 4:**
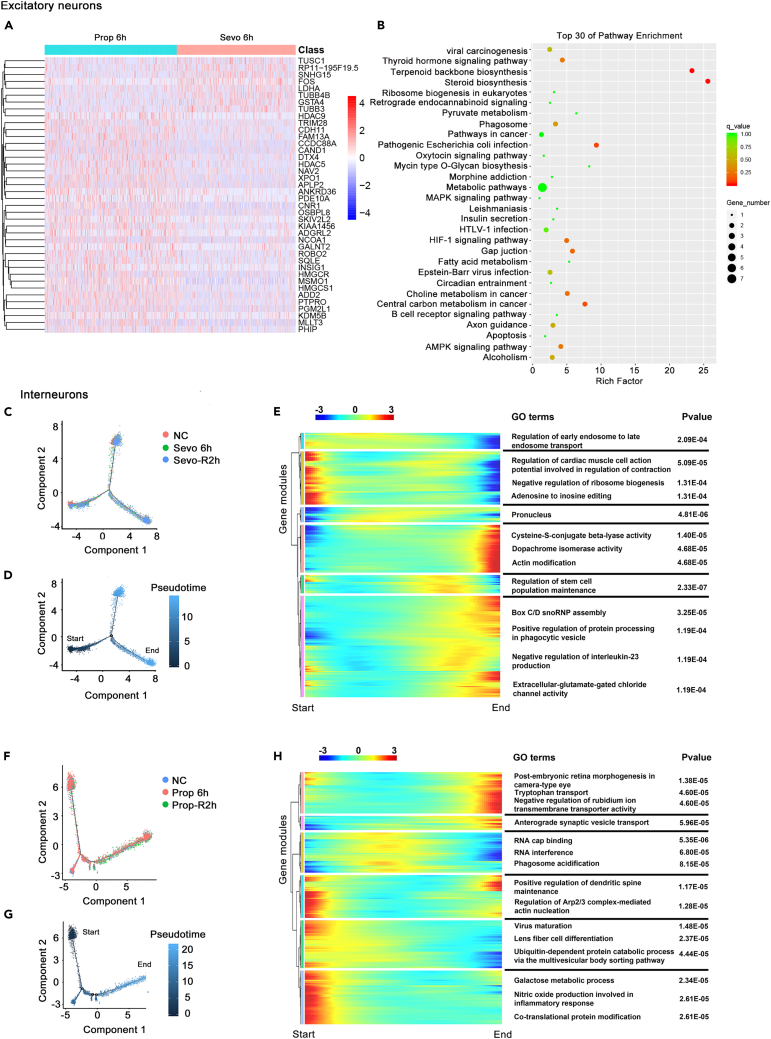
Differentially expressed genes (DEGs) analysis and single-cell trajectory analysis of neurons treated with sevoflurane or propofol for 6h with 2h recovery (A) Heatmap showing differentially expressed genes in excitatory neurons from after 6h of propofol treatment (Prop 6h) and after 6h of sevoflurane treatment (Sevo 6h). Red, upregulation; blue, downregulation. (B) Enriched pathways by DEGs analysis in excitatory neurons from Prop 6h and Sevo 6h treatment. (C) Pseudo-time series analysis results of Interneurons from NC, Sevo 6h, and sevo-R2h groups. (D) Monocle pseudo time series analysis of NC, Sevo 6h, and sevo-R2h groups. (E) Bifurcation of gene expression from start to end (from NC to sevo-R2h) is clustered hierarchically into six modules. Gene ontology analysis of each module reflected the pathways/processes within interneurons affected by sevoflurane. In this heatmap, columns are points in pseudo-time, rows are genes, and the left is the beginning of pseudo-time. Red, upregulation; blue, downregulation. (F) Pseudo time series analysis results of interneurons from group NC, prop 6h, and prop-R2h groups. (G) These three groups from start to end by monocle pseudo time series analysis. (H) Bifurcation of gene expression along from start to end is clustered hierarchically into six modules. Gene ontology analysis of each module reflected the pathways/processes within interneurons affected by propofol. In this heatmap, columns are points in pseudo-time, rows are genes, and the left is the beginning of pseudo-time. The start point of the heatmap is the same as the pseudo time series analysis. Red, upregulation; blue, downregulation.

The additional scRNA sequencing analysis was done to directly compare the differential effects between sevoflurane and propofol on five types of brain cells, as well as enrichment analysis of the top 30 signal pathways. In excitatory neurons, a stark contrast between sevoflurane and propofol is evident, for example *CNR1* and *NAV2* were enhanced by propofol only, whereas FOS was upregulated by sevoflurane (Figure 4A). Similar to the data reported in Figures 3B and 3D, the HIF-1 signaling pathway, axon guidance, and AMPK signaling pathway were enriched (Figure 4B).

### Pseudo-time-trajectory of brain cells unaffected by either anesthetic

The pseudo-time trajectory analysis on scRNA-seq data from brain cells exposed to sevoflurane or propofol demonstrated that neither anesthetic altered the single-cell trajectories^20^^,^^21^ on five different brain cell types throughout the 6 h exposure plus recovery period (Figures 4C, 4D, 4F and 4G and S7-S9). As exemplified by interneurons, despite the substantial expression pattern changes identified in the heatmaps of enriched ontology terms (Figures 4E and 4H), those changes were not associated with significant cellular deviations from the control state. These may suggest that either propofol or sevoflurane did not considerably disrupt the overall transcriptome of primary human brain cells at the developing stages during and after exposure.

### Partial unique effects of general anesthetics on ion channels and calcium transient

**Figure 5:**
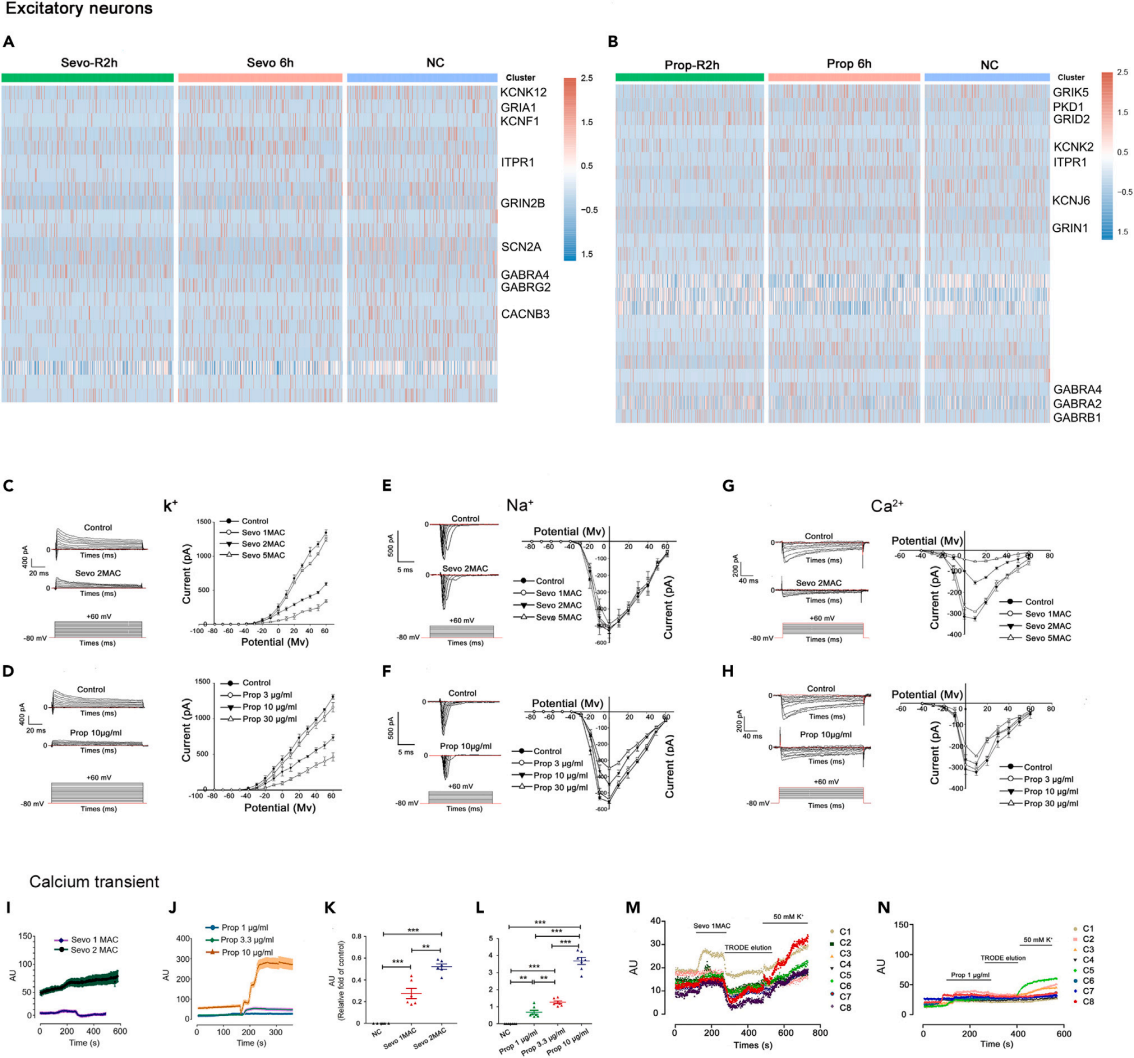
Effects of sevoflurane and propofol on ion channel genes, electrophysiology and calcium transient in PFC neurons (A) Heatmap showing differentially expressed genes in excitatory neurons from NC, Sevo-6h, and Sevo-R2h on ion channel. Red, upregulation; blue, downregulation. (B) Heatmap showing differentially expressed genes in excitatory neurons from NC, Prop 2h, and Prop-R2h on ion channel. Red, upregulation; blue, downregulation. (C and D) Left, representative potassium trace recorded from −80 to +60 mV under control, sevoflurane (2 MAC) or propofol (10 μg/mL) conditions. Right, IV (current-voltage curve) showing the inhibitory effects of increasing concentration of sevoflurane (1, 2, 5 MAC) or propofol (3, 10, 30 μg/mL) on transient outward potassium currents. Compared with the control, the activation threshold potential for I_K_ (delayed rectifier potassium current) did not change significantly (−40 mV), and the IV curve was suppressed significantly with rising concentration of sevoflurane or propofol (p < 0.05; n = 18 neurons from three independent replicates). MAC, minimum alveolar concentration. (E and F) Left, representative Na^+^ trace recorded from −80 to +60 mV under control, sevoflurane (2 MAC) and propofol (10 μg/mL) conditions. Right, IV showing the relationships of voltage-gated Na^+^ channel currents before and after the application of sevoflurane (1, 2, 5 MAC) and propofol (3, 10, 30 μg/mL) in cultured cortical neurons. Compared with the control, the activation threshold potential of Na^+^ in the three groups did not change significantly (−40 mV); sevoflurane did not alter the IV curve (p > 0.05), whereas propofol dose-dependently flattened the IV curve (p < 0.05; n = 18 neurons from three independent replicates). (G and H) Left, representative Ca^2+^ channel IV curves recorded from −80 to +60 mV under negative control, sevoflurane (2 MAC), and propofol (10 μg/mL) conditions. Right, IV showing the relationships of voltage-gated Ca^2+^ channel currents before and after the application of sevoflurane (1, 2, 5 MAC) and propofol (3, 10, 30 μg/mL) in cultured cortical neurons. Compared with the control, the activation threshold potential of Ca^2+^ in the three groups did not change significantly (−40 mV), and the IV curve decreased significantly with the increase of sevoflurane and propofol concentration (p < 0.05), respectively (n = 18 neurons from three independent replicates). (I and J) Representative tracings of [Ca^2+^] from 7 neurons of 3 different batches, before and during exposure to sevoflurane (1 and 2 MAC) or propofol (1, 3.3 and 10 μg/mL). [Ca^2+^]i, intracellular calcium concentration. (K and L) Sevoflurane or propofol at different concentrations induced concentration-dependent increase of [Ca^2+^]i in neurons; Mean ± SD (n = 6-8 neurons from three independent replicates); ∗∗p < 0.001; ∗∗∗p < 0.0001. (M and N) The addition of sevoflurane (1 MAC, up) or propofol (1 μg/ml, down) significantly enhanced the [Ca^2+^]i signal. [Ca^2+^]i signal decreased after elution with TRODE solution. Following the addition of 50mMKCl, [Ca^2+^]i signal was significantly enhanced, indicating normal cell membrane function and satisfactory cell state. Mean ± SD (n = 68 neurons from three independent replicates).

Gene set enrichment analysis from 5 types of cells further identified a total of 341 human ion channel-related genes that responded to anesthetics including those encoding the subunits of voltage-gated Na^+^, K^+^, Ca^2+^ ion channels, ligand-gated NMDA, AMPA, and GABA receptor. It can be readily appreciated that many of those subunits are putative binding targets of general anesthetics including the GABA_A_R receptor subunit-alpha1, -alpha2, -beta1, -beta2, and -gamma1 encoded by *GABRA4*, *GABRA2, GABRG2, and GABRB1*; all of which exhibited immediate and reversible transcriptional perturbations in response to sevoflurane or propofol exposure (Figures S10 and S11). As exemplified by the excitatory neurons, propofol and sevoflurane differentially regulated ion channel-related genes. For example, sevoflurane altered the expression of *KCNK12, GRIA1,* and *KCNF1* (K^+^ ion channels-related genes), *ITPR1, GRIN2B,* and *CACNB3* (Ca^2+^ ion channels-related genes) and *SCN2A* (Na^+^ ion channels-related gene), which are distinct from propofol-associated transcriptional changes in *GRIK5, KCNK2,* and *KCNJ6* (K^+^ ion channels-related genes), *PKD1, ITPR1* and *GRIN1* (Ca^2+^ ion channels-related genes) and *GRID2* (Na^+^ ion channels-related gene) (Figures 5A and 5B).

Sevoflurane suppressed both the outward K^+^ current and the inward Ca^2+^ current in a concentration-dependent manner, whereas an increasing propofol concentration effectively blocked the inward Na^+^ current. The inhibitory effect of sevoflurane on K^+^ and Ca^2+^ current appeared to be more pronounced than that of propofol (Figures 5C-5H). This is in line with our previous enrichment of related genes, K^+^ and Ca^2+^ ion channel genes were inhibited after sevoflurane and propofol treatment (e.g., *KCNK12*, CACNB3, and ITPR1) while Na^+^ channel genes change less (e.g., SCN2A and GRID2) (Figures 5A and 5B). Our enrichment analysis also revealed extensive gene expression changes to ion channel subunits during anesthetic exposure and recovery phase (e.g., sodium voltage-gated channel alpha subunit 2/SCN2A, calcium voltage-gated channel subunit alpha 1A/CCNA1A, potassium voltage-gated channel subfamily D member 2/KCND2 (Figures 5A, 5B, and S10).

Furthermore, we found that propofol and sevoflurane led to transient, dose/concentration-dependent increase of intracellular calcium concentration (Figures 5I-5L), which rapidly returned to the baseline level when anesthetics were eluted without intracellular calcium overload (Figures 5M and 5N). This provides evidence that sevoflurane/propofol administered at clinically relevant doses do not disrupt intracellular calcium homeostasis and may be safe for developing human excitatory neurons. However, the gene enrichment heatmap (Figure S10) identified increasing gene expressions related to inositol 1,4,5-triphosphate receptor (*ITPR1/ITPR2*) and ryanodine receptor (*RYR1/RYR2/RYR3*), which are.

**Figure 6:**
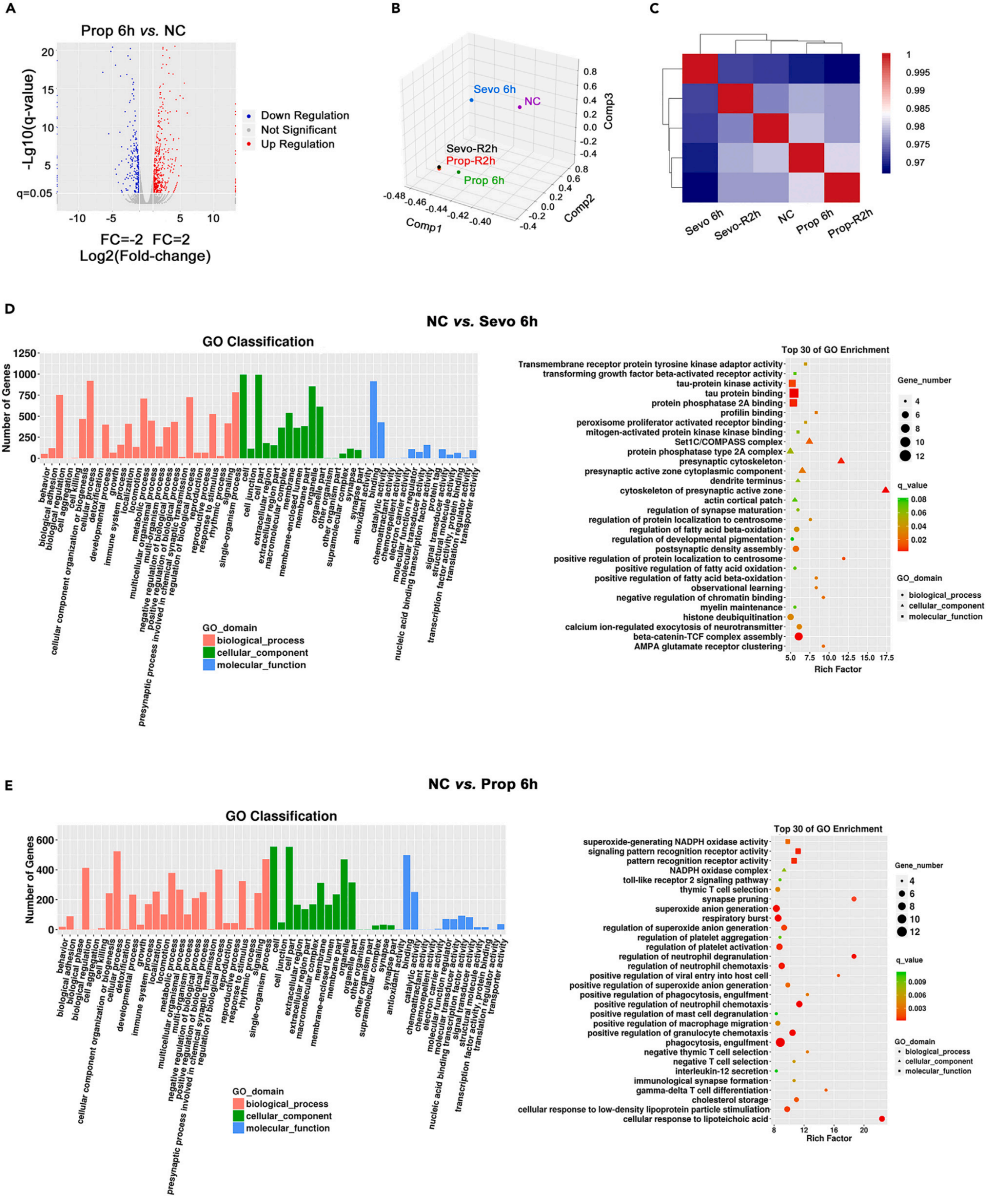
RNA-seq results of NC, Sevo 6h, Prop 6h, Sevo-R2h, and Prop-R2h groups (A) Differential gene volcano map between Prop6h and NC. Red, up-regulated and blue, down-regulated. (B) Principal component analysis (PCA) images of samples based on gene expression. Although the NC group was treated with sevoflurane or propofol for 6h and changed in two different directions, the gene expression in both groups was almost the same after 2h recovery. (C) Heatmap of differential genes from five treatment groups. The amount of genetic difference between each group. Red, upregulation; blue, downregulation. (D) Left, a statistical map of GO (gene ontology) functional classification of differentially expressed genes of NC and Sevo 6h groups. Red, biological process. Green, cellular component. Blue, molecular function. Right, the top 30 signal pathways of GO enrichment distribution points showing differentially expressed genes in NC and Sevo 6h groups. (E) Left, a statistical map of GO (gene ontology) functional classification of differentially expressed genes of NC and Prop 6h groups. Red, biological process. Green, cellular component. Blue, molecular function. Right, the top 30 signal pathways of GO enrichment distribution points showing differentially expressed genes of NC and Prop 6h groups.

Conventional RNA sequencing analysis also revealed that single exposure of propofol or sevoflurane was associated with substantial transcriptional alterations in the PFC mixed culture (Figures 6A-6E). Interestingly, the overall gene expression profile at 2 h recovery after sevoflurane or propofol became indistinguishable (Figure 6B) albeit substantial divergence in gene expression pattern during the 6 h exposure to either anesthetic. Taken together, the data from scRNA-seq and conventional RNA sequencing with the PFC mixed cells demonstrated that sevoflurane and propofol-induced significant, differential, and temporal changes to gene expression across diverse ontology terms in all five cell types, and the brain cells were capable of “self-normalising”/reversing such changes after the removal of general anesthetics (Figures S12-S15).

## Discussion

Our single-cell-RNA sequencing study, for the first time, illustrated the comprehensive and diverse gene responses in different human PFC cell types following a single exposure to general anesthetics. Both propofol and sevoflurane at clinically relevant dose/concentration promoted “microgliosis” but only sevoflurane changed microglia gene similarity. Propofol and sevoflurane each extensively but transiently altered transcriptome profiling across microglia, excitatory neurons, interneurons, astrocytes, and oligodendrocyte progenitor cells. Within the excitatory neurons and microglia, exemplary ion-gated and ligand-gated ion channels related genes responsive to either anesthetic included *SCN1A, CACNAB2, KCNA1, GABRR2* and *GRINA1* amongst many others.

Both propofol and sevoflurane at clinically relevant dose/concentration promoted “microgliosis” but only sevoflurane changed microglia transcriptional similarity. In line with our findings, sevoflurane was reported to aggravate microglia-mediated neuroinflammation via the downregulation of PPAR-γ in the hippocampus.^22^ Collectively, our data showed that single exposure to general anesthetics does not alter cell number and similarity for most types of fetal PFC cells except microglia. Propofol and sevoflurane each extensively but transiently altered transcriptome profiling across microglia, excitatory neurons, interneurons, astrocytes, and oligodendrocyte progenitor cells. Of particular interest, sc-RNA seq heatmaps highlighted a large number of previously unidentified genes highly responsive to sevoflurane or propofol, exemplified by *JAKMIP1*, *RBM4B,* and *THY1*, to represent potential molecular targets through which general anesthetics impart amnesia, analgesia and loss of consciousness. Interestingly, only propofol increased *CNR1* expression in excitatory neurons which may result in increasing brain endocannabinoid.^23^^,^^24^

Recently, repeated exposure to sevoflurane in neonatal mice induced cognitive and behavioral deficits which was associated with the hippocampus transcriptome changes.^25^ Yamamoto et al. further examined the differential gene expression across PFC, striatum, hypothalamus, and hippocampus in sevoflurane-anesthetised mice and found that sevoflurane upregulated angiogenesis and downregulated brain cell differentiation-related genes.^26^ Moreover, the dysregulated genes/pathways associated with sevoflurane exposure were also reported in rhesus macaques.^27^ However, the work reported here was the first-line evidence on transcriptome changes in human fetal prefrontal cortex cells following general anesthetic exposure using scRNA sequencing technique, providing accurate identification of gene expression patterns within each cell type. Unlike enrichment of *KLF4* and *NFATC2* in animals (Yamamoto et al., 2020; Cheng et al., 2022), our study identified enrichment in KLF6, KLF10, and NFATC4 in human PFC cells that could be due to species variation.

We, for the first time, determined the effects of general anesthetics on sodium, potassium, and calcium currents from primary human excitatory neurons. Our findings are consistent with previous studies demonstrating a reduction in peak amplitude of potassium currents following sevoflurane exposure in xenopus oocytes.^28^ In contrast to other studies showing that sevoflurane activated voltage-gated K^+^ channels and increased outward K^+^ current in the pons and cerebellar granule neurons in rats,^7^ suppression of outward K^+^ current following sevoflurane or propofol exposure was found in our study. This difference was likely due to that human prefrontal cortex neurons behave differently to other species. Within the excitatory neurons and microglia, exemplary ion-gated and ligand-gated ion channels related genes responsive to either anesthetic included *SCN1A, CACNAB2, KCNA1, GABRR2,* and *GRINA1* amongst many others. These findings suggest that sevoflurane may have multiple mechanisms of action on brain cells when compared to propofol. This is corroborated with other studies; *in vitro* studies with spinal neurons showed a complete inhibition of action potential activity with sevoflurane but not propofol.^29^ Furthermore, metabolomic profiling studies of parietal cortex of children’s brains revealed that sevoflurane exposure led to higher glucose consumption and lactate generation than propofol.^5^ This suggested greater glycolysis, glutamate-neurotransmitter cycling and lactate shuttling between astrocytes and neurons following sevoflurane exposure.^5^ Compelling evidence demonstrated that propofol acts upon gamma-aminobutyric acid type A (GABAA) receptors whereas the contribution of glycine receptors remains uncertain.^29^^,^^30^ Volatile anesthetic sevoflurane also likely exerts its central depressant effect through the glycine and GABAA receptors, but indirect evidence suggests a mechanism of action distinct from propofol.^29^ In the present study, we found that propofol and sevoflurane exposure were associated with significant, distinct gene expression alterations across an extensive list of ion channel-related genes, such as *SCN8A*, *CACNB4*, *KCNJ3*, *GLRA2* and *GABARA5* (Figures 5A and 5B). In addition, we also disclosed a large pool of previously unidentified ion channel/receptor subunits that may represent novel molecular targets of anesthetics. Overall, it is yet to be determined whether the perturbations to ion channel subunit expression on single cell level found in our study are accountable for the transient inhibitory effects of anesthetics on ion channel currents and neuronal excitability.

### Limitations of study

Our study is not without limitations. First, brain cell cultures were used and this *in vitro* setting cannot fully recapitulate in *in vivo* setting and humans. Second, it is unclear to what extent the findings derived from fetal PFC cells can be extrapolated to the adult human brain. However, it should be pointed out that adult human brain cells cannot be cultured for the study type reported herein. Nevertheless, our study is the first one that clearly demonstrated the distinct and significant effects of general inhalational versus intravenous anesthetics on human brain cells at the gene transcriptome, molecular and cellular levels.

The distinct changes to microglial population and reactivity by both anesthetics are of significance, with studies showing that sevoflurane promoted neurofunctional impairment through glial-mediated neuroinflammation,^31^ and that propofol inhibited microglial activation to protect traumatic brain injury.^32^ Anesthetics have also been shown to increase cancer cell growth,^33^ protect neuronal and other type cell death,^34^ or even trigger neuronal cell death.^35^^,^^36^ However, our data likely only represent a “snapshot” of the actions of general anesthetics on the human brain cells. Whether transcriptome alterations translate into mid-to long-term phenotypic and functional modifications needs to be addressed in the future. Nonetheless, the plethora of transcriptome changes and pathway enrichment identified by sc-RNA seq reported here may serve as a robust database for future mechanistic studies into general anesthetics.

## STAR★Methods

### Key resources table

**Table:** 

REAGENT or RESOURCE	SOURCE	IDENTIFIER
**Antibodies**		

Tuj1	Merck Millipore	RRID:AB_580035
MAP2	Abcam	RRID:AB_776174
GFAP	Cell Signaling Technology	RRID:AB_2631098
HIF-1α	Novus Biologicals	RRID:AB_10001154
CNR1	Invitrogen	RRID:AB_10854442
HRP-conjugated donkey anti-rabbit	Sigma	RRID:AB_92591

**Biological samples**		

human fetal prefrontal cortex	People’s Hospital of Zhengzhou University, Henan, China	N/A

**Chemicals, peptides, and recombinant proteins**		

Hibernate^TM^-E medium	Invitrogen	Cat# A1247601
streptomycin	Solarbio	Cat# P1400
EDTA	Solarbio	Cat# T1300
paraformaldehyde	Sigma	Cat# P1524
Dulbecco’s Modified Eagle Medium	Hyclon	Cat# SH30022.01
foetal bovine serum	Gbico	Cat# 10099141C
Neurobasal	Gbico	Cat# 21103049
sevoflurane	Jiangsu Hengrui Medicine, China	Cat# 22101231
Propofol	AstraZeneca, UK	Cat# N01AX10
paraformaldehyde	Biosharp Life Sciences	Cat# BL539A
triton X-100	Solarbio Life Science	Cat# T8200
luminol reagent	Santa Cruz	Cat# sc-2048

**Deposited data**		

Raw and analyzed data	This paper	GEO: GSE196239

**Software and algorithms**		

ImageJ	Imagej	https://wsr.image.net/distros/win/ij 153-win-java8.zip
Graphpad Prism	Graphpad	https://www.graphpad.com/scientific-software/prism/
Cell Ranger v3.1.0 Single-Cell	10X Genomics	https://www.10xgenomics.com/

### Resource availability

#### Lead contact

Further information and requests for resources and reagents should be directed to and will be fulfilled by the lead contact, Professor Jiaqiang Zhang zhangjiq@zzu.edu.cn.

#### Materials availability

This study did not generate new unique reagents.

### Experimental model and subject details

#### Ethics statement

The experimental protocol was built up according to the International Society for Stem Cell Research guidelines for foetal tissue donation and approved by the Ethics Committee of People’s Hospital of Zhengzhou University (2019-127), Henan, China and registered with the Chinese Clinical Trial Registry (www.chictr.orG.cn; ChiCTR2000029557). Written informed consent was obtained from foetal tissue donors after pregnant women voluntarily gave up the pregnancy due to embryo abnormal development (e.g., severe cardiac malformations, serious genetic diseases and chromosomal abnormalities, but no abnormalities of the brain). Then, the foetal cortical tissue samples were collected accordingly and only used for the current study.

#### Tissue sample collection and culture

PFC tissue from four fetuses were processed and were subsequently hybridized to probe sets and pooled with four samples in a single lane of a Chromium chip. The tissues were collected and dissected in ice-cold Hibernate^TM^-E medium (Invitrogen, A1247601) with penicillin and streptomycin (Solarbio, P1400). The PFC tissues were chopped into small pieces with a scalpel blade and digested in 0.25% trypsin-EDTA (Solarbio, T1300) at 37°C for 10 min. The cell suspension was further pipetted to be dispersed to single cells, then the cells were passed through 70 μm filter and centrifuged at 1000 rpm for 7 min at 4°C. The supernatant was aspirated, and the cell pellet was resuspended and seeded to 6-well plates, covered with 20 μg/ml paraformaldehyde (PDL) (Sigma, P1524), in Dulbecco’s Modified Eagle Medium (DMEM) (Hyclon, SH30022.01) with 10% foetal bovine serum (FBS) (Gbico, 10099141C). After 4 hrs incubation, the medium was aspirated, and the cells were cultured in Neurobasal medium (Gbico, 21103049) supplemented with 2% B27. Every three days half of the medium was replaced by fresh Neurobasal medium.^19^^,^^37^^,^^38^

### Method details

#### Anesthetic exposure, immunofluorescence, single cell RNA-seq and immunoblotting analysis

Cells were divided into 5 groups: Naive control (NC, collected at 8 hrs); Sevoflurane treatment for 6 hrs (Sevo 6h) and recovery for 2 hrs (Sevo-R2h); Propofol treatment for 6 hrs (Prop 6h) and recovery for 2 hrs (Prop-R2h). The cultured cells were incubated in a 1.5 L airtight temperature-controlled chambers with inlet and outlet valves and an internal electric fan. For sevoflurane (Jiangsu Hengrui Medicine, China) delivery, the chamber inlet was connected to a closed gas delivery system consisting of a calibrated oxygen flow metre and an inline sevoflurane vaporiser (Abbott Laboratories, Maidenhead, UK). The outlet gas was monitored (Datex gas monitor, Helsinki, Finland) to ensure the sevoflurane in chamber reach the target concentration (3.3%),^39^ the equivalent of 1.0 minimum alveolar concentration, MAC). Cells were then incubated in the sealed chamber for 6 hrs at 37°C.^40^ In the propofol group, the PFC cells were treated with 20 μΜ propofol (AstraZeneca, UK) for 6 hrs (the equivalent of half maximal effective concentration *in vitro*).^41^^,^^42^ An exposure duration of 6 hrs and the anesthetic concentrations were chosen in accordance with previous studies and reflect the concentration and duration for the longest paediatric anesthesia.^43^ In any groups either during anesthetic or mock exposure or recovery phase, all cells were treated under identical conditions except with or without anesthetics and exposed to the identical mixture gases of 20% oxygen, 5% carbon dioxide and balanced with nitrogen at 37°C.

The cells isolated from foetal prefrontal cortex were washed in PBS for twice, then followed by 4% paraformaldehyde (Biosharp Life Sciences, BL539A) for 15min. After fixing the cells, the 0.3% triton X-100 (Solarbio Life Science, T8200) were added to the cells to permeate the cell membrane. The penetrated cells were pre-incubated in 5% normal goat serum for 1 h at room temperature, followed by overnight incubation with primary antibody Tuj1 (MAB1637, Merck Millipore, 1:200), MAP2(ab32454, Abcam, 1:200) and GFAP (12389S, CST, 1:200) at 4°C. After sufficient washing, cells were incubated with the appropriate secondary antibody conjugated with Alexa488 and Alexa 594 (Jackson, 1:200) for 1 h at room temperature. After washing, the samples were analyzed on Olympus microscope.

Cell Ranger v3.1.0 Single-Cell Software suite from 10X Genomics platform was used for gene comparison. Genes expressed fewer than three cells in a sample were excluded. Cells expressed fewer than 200 genes or cells’ mitochondrial gene content > 25% of the total UMI count were also excluded. Unsupervised clustering analysis of gene expression were performed by the Seurat package.^44^ Cells were clustered using Louvain method after computing a shared nearest neighbour graph.^44^ For each cluster, Wilcoxon Rank-Sum Test was used for gene expression comparison. SingleR41 and gene markers were used to identify cell type. For the cell similarity calculation, variable genes of target cells were selected using FindVariableGenes function of Seurat (dispersion cutoff 1). The average gene expression profile of a cell population was calculated using previous genes as the centre. The Pearson correlation coefficient of each cell to the centre was calculated and shown as a plot.^45^ The Monocle package^20^^,^^46^^,^^47^ was used to analyse single-cell trajectories.^48^ Pseudotime trajectory analysis on single-cell transcriptomics was achieved by determining the pattern of a dynamic process experienced by cells and then arrange cells based on their progression through the process. “DDRTree” was applied to reduce dimensions and the visualisation functions. “Plot_Cell_Trajectory” or “Plot_Complex_Cell_Trajectory” were used to plot the minimum spanning tree on cells. The R package’s clusterProfiler was applied to identify a priori-defined gene sets that showed statistically significant differences between given clusters.^49^

Cytarabine (Ara-C, sigma,147-94-4, 10μmol/L) was used to inhibit the growth of glial cells to obtain a neuron-enriched culture. Protein content of neuronal lysates was quantified using Bradford Protein Assay (Bio-rad) against known concentrations of bovine serum albumin (Bio-rad). Protein samples were prepared with 4X SDS sample buffer (Bio-rad) and were resolved by SDS-PAGE, before transfer onto PVDF membranes (Bio-rad). Membrane was incubated with HIF-1α antibody (Novus Biologicals, NB100-105, 1:1000) or CNR1 antibody (Invitrogen, BS-1683R,1:1000) overnight at 4C, and was further probed with HRP-conjugated donkey anti-rabbit antibody (Sigma, AP510, 1:5000) on day2 for 1hr at room temperature. Protein bands were developed by chemiluminescence luminol reagent (sc-2048, Santa Cruz).

#### Anesthetic treatment and electrophysiology

Whole-cell currents were recorded using Axopatch 200B patch-clamp amplifier interfaced to Digidata 1440A (Axon Instruments, USA) at room temperature (20-22°C) under Olympus inverted microscope (IX71, Japan). Propofol was diluted to 3, 10, and 30 μg/ml (approximately 17, 56, and 168 μΜ). Sevoflurane was diluted from saturated aqueous stock solutions to 4-6 mM (the equivalent of 10 MAC) into airtight glass syringes 12-18 h before the experiments. The supersaturated solution of sevoflurane was diluted to 0.46, 0.92, and 2.30 μΜ (the equivalent of 1 MAC, 2 MAC, and 5 MAC).^3^^,^^40^^,^^50^^,^^51^ Sevoflurane and propofol were applied to attached cells using an ALA-VM8 pressurised perfusion system (ALA Scientific, Westbury, NY) through a perfusion pipette, positioned 100 μm away from patched cells. The concentration of sevoflurane was determined by the perfusate at the tip of the pipette and analyzed by gas chromatography. For whole-cell recording of the voltage-gated Na^+^ channel and the voltage-gated K^+^ channel, the bath solution had the following composition (in mM): NaCl 140, KCl 5, CaCl_2_ 2, CdCl_2_ 0.3, HEPES 10, glucose 10, and the pH was adjusted to 7.4 with NaOH. The pipette solution contained (in mM): K-gluconate 140, KCl 5, CaCl_2_ 1, MgCl_2_ 2, HEPES 10, EGTA 11, K_2_-ATP 2, GTP 0.1, and the pH was adjusted to 7.2 with KOH. The cells were clamped at - 80 mV and depolarized from - 80 mV to + 60 mV with 10 mV steps during 100 ms. For whole-cell recording of the voltage-gated Ca^2+^ channel, the bath solution had the following composition (in mM): Choline-chloride 110, TEA-Cl 20, 4-AP 10, BaCl_2_ 10, MgCl_2_ 1, HEPES 10, glucose 10, TTX 0.001, and the pH was adjusted to 7.4 with NaOH. The pipette solution contained (in mM): CsCl 140, KCl 5, MgCl_2_ 1, HEPES 10, EGTA 10, K_2_-ATP 2, GTP 0.1, and the pH adjusted to 7.2 with CsOH. The cells were clamped at - 80 mV and depolarized from - 40 mV to + 60 mV with 10 mV steps during 200 ms. Data were analyzed using Clampfit software (version 10.0; Axon instrument) and GraphPad prism (v.6.0; GraphPad software).

#### Anesthetic exposure and calcium transients

The [Ca^2+^] ions in the cultured neurons were measured under fluorescence inverted microscope system (Olympus, Tokyo, Japan) with Fura-2-AM fluorescence (with an emission of 510 nm), which is a cytoplasmic dye used to mark cytoplasmic changes in calcium, was chosen as the [Ca^2+^] ion fluorescent indicator. Neurons were identified morphologically by their cell body and synapses before they were studied, and the slit aperture was focused on the cell body. Neurons were exposed to the supersaturated solution of sevoflurane at 0.46 and 0.92 μΜ (the equivalent of 1 and 2 MAC) or propofol at a standard of 1, 3.3, and 10 μg/ml (approximately 6, 18, and 56 μΜ) and fluorescence images were captured using a Photometrics CoolSNAP HQ2 CCD monochrome Camera (Roper Scientific, Optical Apparatus Co.) during anesthetic exposure and being washed off phase. Illumination was provided by a Lambda DG-4 (Sutter Instrument Company, Novato, CA) with exposures in the range of 200-300 ms. Data were collected and analyszed with Meta Flour analysis software.^9^^,^^52^

### Quantification and statistical analysis

Data were presented as the mean ± SD. The n number referred to the number of cells studied. Data were analyzed with Analysis of Variance (ANOVA) followed by *post hoc* Newman–Keuls test for comparison or with the paired student t-tests where appropriate. Linear regression analysis (JMP; SAS Institute, Cary, NC) was performed to test for a dose-dependent effect of the increase of [Ca^2+^] ions. A *P* value less than 0.05 was considered to be of statistical significance.

### Additional resources

The protocol and experimental procedures were in compliance with the Regulations on Administration of Human Genetic Resources, governed by the Chinese Ministry of Health.

## Data Availability

• All data reported in this paper can be shared by the lead contact on request. • This article does not report original code. • Any additional information required to re-analyze the data reported in this paper is available from the lead contact upon request. • The single-cell RNA-seq data used in this study have been deposited in the Gene Expression Omnibus (GEO) under the accession number GSE196239. Raw image files used in the figures that support the findings of this study are available from the corresponding authors upon reasonable request.
