# Supplementary material for: General anesthetic action profile on the human prefrontal cortex cells through comprehensive single-cell RNA-seq analysis
Source: iScience. 2023 Mar 31;26(4):106534. doi: 10.1016/j.isci.2023.106534 (PMC10130912; doi:10.1016/j.isci.2023.106534)
Supplement: Document S1. Figures S1-S15 and Table S1 [file mmc1.pdf]

iScience, Volume *26*

Supplemental information

General anesthetic action profile on the human prefrontal cortex cells through comprehensive single-cell RNA-seq analysis

Enqiang Chang, Yangyang Wang, Ruilou Zhu, Lingzhi Wu, Yitian Yang, Shuang Zeng, Ningtao Li, Xiaoguo Ruan, Mingyang Sun, Wei Zhang, Jun Zhou, Mengrong Miao, Hui Zhi, Hailin Zhao, Qian Chen, Qizhe Sun, Emer Chang, Albert Chang, Tingting Zhang, Xinfang He, Kan Liu, Songhua Ma, Weizhong Zhu, Youming Zhang, Luca Magnani, Daqing Ma, and Jiaqiang Zhang

Excitatory neurons

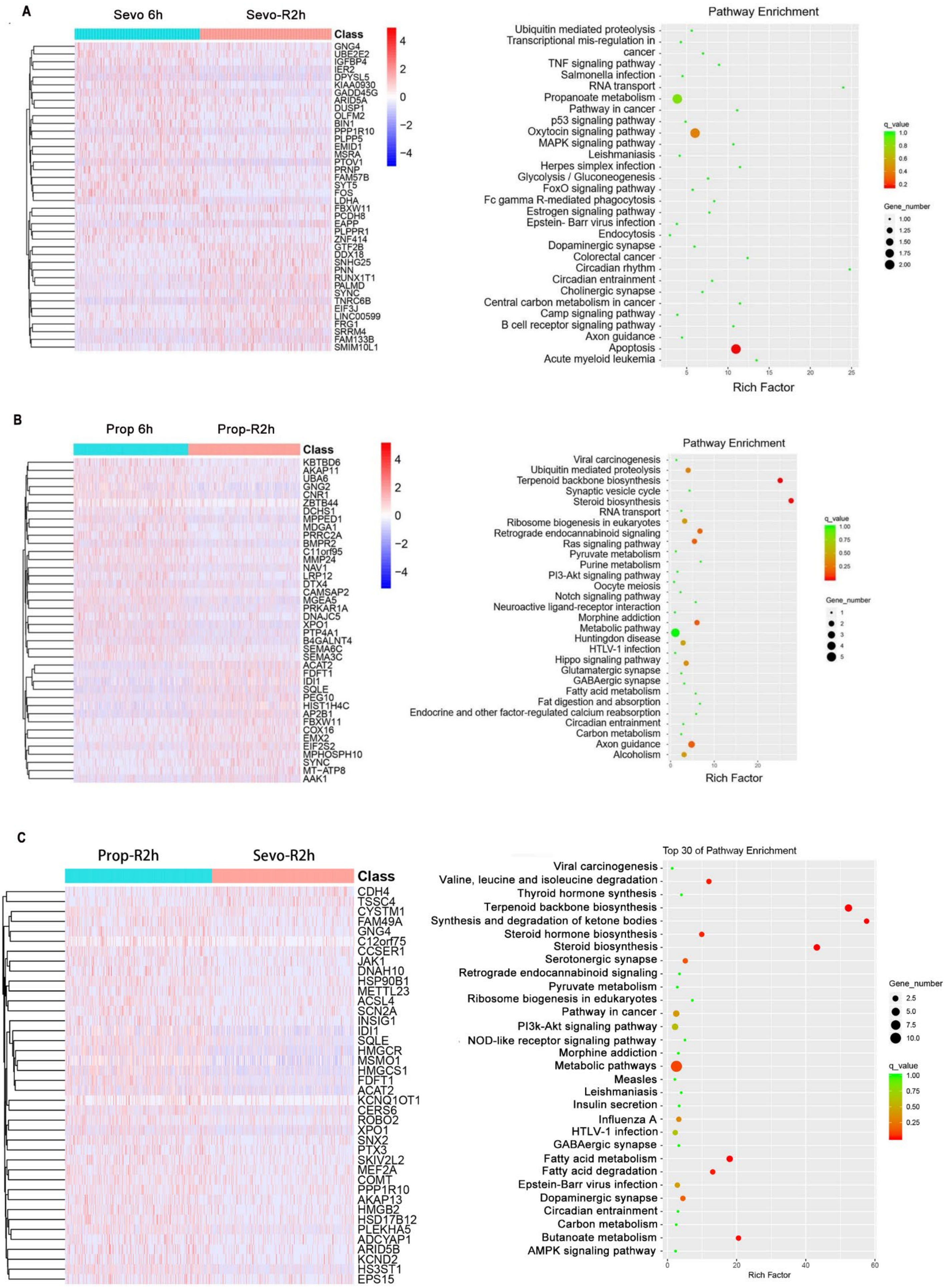

Figure S1. Differential expressed genes (DEGs) analysis and enriched pathways analysis of excitatory neurons, Related to Figure 3. Red, upregulation; blue, downregulation.
A. Left, Heatmap showing differentially expressed genes in excitatory neurons from Sevo 6h and Sevo-R2h groups. Red, increase, blue, decrease. **Right,** Enriched pathways analysis in excitatory neurons from Sevo 6h and Sevo-R2h.
B. Left, Heatmap shows differentially expressed genes in excitatory neurons from Prop 6h and Prop-R2h. Red, increase, blue, decrease. **Right,** Enriched pathways by DEGs analysis in excitatory neurons from Prop 6h and Prop-R2h.
C. Left, Heatmap shows differentially expressed genes in excitatory neurons from Prop-R2h and Sevo-R2h. Red, increase, blue, decrease. **Right,** Enriched pathways by DEGs analysis in excitatory neurons from Prop-R2h and Sevo-R2h.

Astrocytes

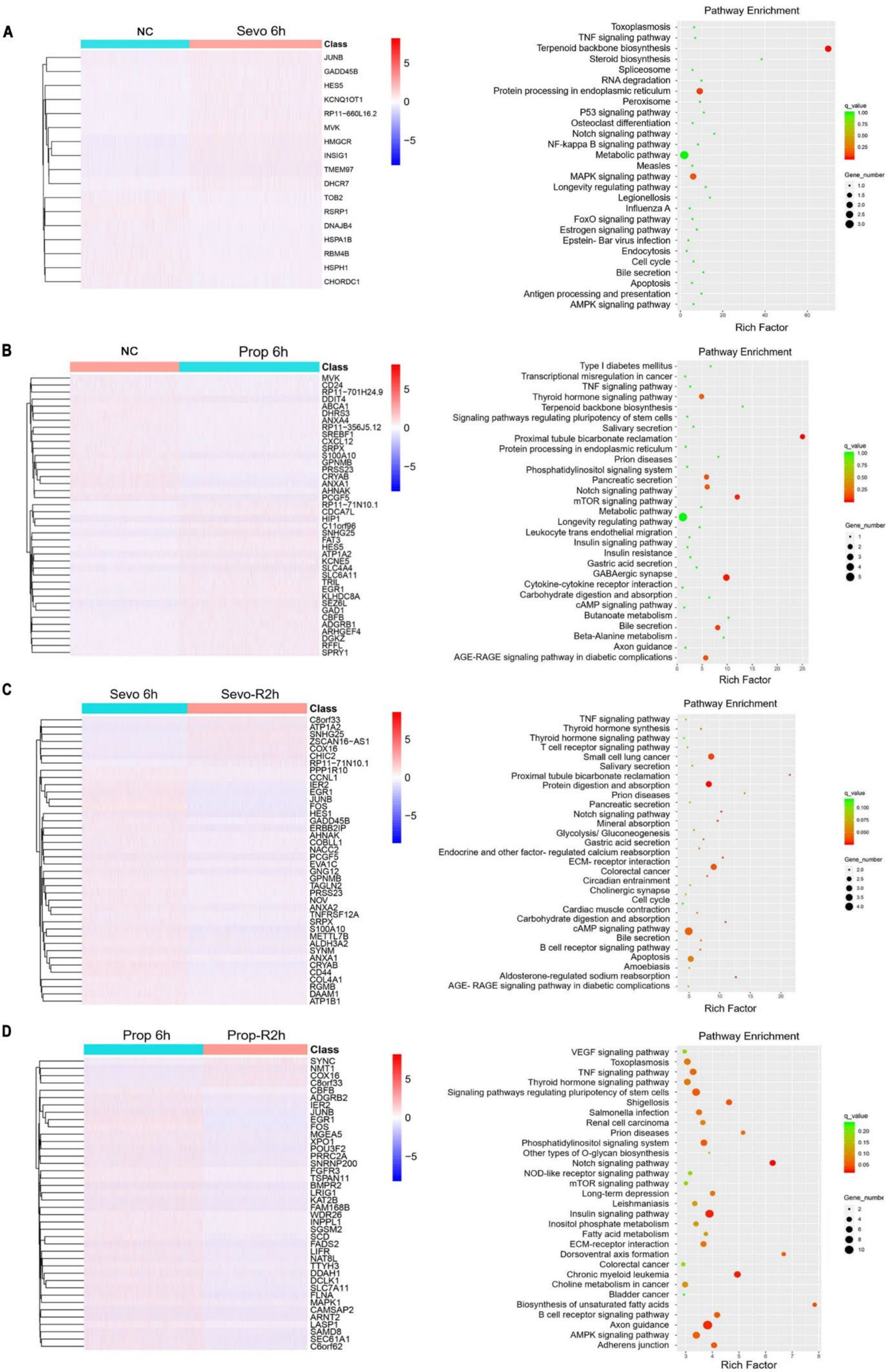
A
NC
Sevo 6h
Class
JUNB
GADD45B
HES5
KCNQ1OT1
RP11−660L16.2
MVK
HMGCR
INSIG1
TMEM97
DHCR7
TOB2
RSRP1
DNAJB4
HSPA1B
RBM4B
HSPH1
CHORDC1
Pathway Enrichment
Toxoplasmosis
TNF signaling pathway
Terpenoid backbone biosynthesis
Steroid biosynthesis
Spliceosome
RNA degradation
Protein processing in endoplasmic reticulum
Peroxisome
P53 signaling pathway
Osteoclast differentiation
Notch signaling pathway
NF-kappa B signaling pathway
Metabolic pathway
Measles
MAPK signaling pathway
Longevity regulating pathway
Legionellosis
Influenza A
FoxO signaling pathway
Estrogen signaling pathway
Epstein- Bar virus infection
Endocytosis
Cell cycle
Bile secretion
Apoptosis
Antigen processing and presentation
AMPK signaling pathway
Rich Factor
q_value
Gene_number
B
NC
Prop 6h
Class
MVK
CD24
RP11−701H24.9
DDIT4
ABCA1
DHRS3
ANXA4
RP11−356J5.12
SREBF1
CXCL12
SRPX
S100A10
GPNMB
PRSS23
CRYAB
ANXA1
AHNAK
PCGF5
RP11−71N10.1
CDCA7L
HIP1
C11orf96
SNHG25
FAT3
HES5
ATP1A2
KCNE5
SLC4A4
SLC6A11
TRIL
EGR1
KLHDC8A
SEZ6L
GAD1
CBFB
ADGRB1
ARHGEF4
DGKZ
RFFL
SPRY1
Pathway Enrichment
Type I diabetes mellitus
Transcriptional misregulation in cancer
TNF signaling pathway
Thyroid hormone signaling pathway
Terpenoid backbone biosynthesis
Signaling pathways regulating pluripotency of stem cells
Salivary secretion
Proximal tubule bicarbonate reclamation
Protein processing in endoplasmic reticulum
Prion diseases
Phosphatidylinositol signaling system
Pancreatic secretion
Notch signaling pathway
mTOR signaling pathway
Metabolic pathway
Longevity regulating pathway
Leukocyte trans endothelial migration
Insulin signaling pathway
Insulin resistance
Gastric acid secretion
GABAergic synapse
Cytokine-cytokine receptor interaction
Carbohydrate digestion and absorption
cAMP signaling pathway
Butanoate metabolism
Bile secretion
Beta-Alanine metabolism
Axon guidance
AGE-RAGE signaling pathway in diabetic complications
Rich Factor
q_value
Gene_number
C
Sevo 6h
Sevo-R2h
Class
C8orf33
ATP1A2
SNHG25
ZSCAN16−AS1
COX16
CHIC2
RP11−71N10.1
PPP1R10
CCNL1
IER2
EGR1
JUNB
FOS
HES1
GADD45B
ERBB2IP
AHNAK
COBLL1
NACC2
PCGF5
EVA1C
GNG12
GPNMB
TAGLN2
PRSS23
NOV
ANXA2
TNFRSF12A
SRPX
S100A10
METTL7B
ALDH3A2
SYNM
ANXA1
CRYAB
CD44
COL4A1
RGMB
DAAM1
ATP1B1
Pathway Enrichment
TNF signaling pathway
Thyroid hormone synthesis
Thyroid hormone signaling pathway
T cell receptor signaling pathway
Small cell lung cancer
Salivary secretion
Proximal tubule bicarbonate reclamation
Protein digestion and absorption
Prion diseases
Pancreatic secretion
Notch signaling pathway
Mineral absorption
Glycolysis/ Gluconeogenesis
Gastric acid secretion
Endocrine and other factor- regulated calcium reabsorption
ECM- receptor interaction
Colorectal cancer
Circadian entrainment
Cholinergic synapse
Cell cycle
Cardiac muscle contraction
Carbohydrate digestion and absorption
cAMP signaling pathway
Bile secretion
B cell receptor signaling pathway
Apoptosis
Amoebiasis
Aldosterone-regulated sodium reabsorption
AGE- RAGE signaling pathway in diabetic complications
Rich Factor
q_value
Gene_number
D
Prop 6h
Prop-R2h
Class
SYNC
NMT1
COX16
C8orf33
CBFB
ADGRB2
IER2
JUNB
EGR1
FOS
MGEA5
XPO1
POU3F2
PRRC2A
SNRNP200
FGFR3
TSPAN11
BMPR2
LRIG1
KAT2B
FAM168B
WDR26
INPPL1
SGSM2
SCD
FADS2
LIFR
NAT8L
TTYH3
DDAH1
DCLK1
SLC7A11
FLNA
MAPK1
CAMSAP2
ARNT2
LASP1
SAMD8
SEC61A1
C6orf62
Pathway Enrichment
VEGF signaling pathway
Toxoplasmosis
TNF signaling pathway
Thyroid hormone signaling pathway
Signaling pathways regulating pluripotency of stem cells
Shigellosis
Salmonella infection
Renal cell carcinoma
Prion diseases
Phosphatidylinositol signaling system
Other types of O-glycan biosynthesis
Notch signaling pathway
NOD-like receptor signaling pathway
mTOR signaling pathway
Long-term depression
Leishmaniasis
Insulin signaling pathway
Inositol phosphate metabolism
Fatty acid metabolism
ECM-receptor interaction
Dorsoventral axis formation
Colorectal cancer
Chronic myeloid leukemia
Choline metabolism in cancer
Bladder cancer
Biosynthesis of unsaturated fatty acids
B cell receptor signaling pathway
Axon guidance
AMPK signaling pathway
Adherens junction
Rich Factor
q_value

Figure S2. Differential expressed genes (DEGs) analysis and enriched pathways analysis of Astrocytes, Related to Figure 3. Red, upregulation; blue, downregulation.

A. Left, Heat map shows differentially expressed genes in Astrocytes from the NC and Sevo 6h. Red, increase, blue, decrease. **Right,** enriched pathways by DEGs analysis in Astrocytes from the NC and Sevo 6h.

B. Left, Heat map shows differentially expressed genes in Astrocytes from the NC and Prop 6h. Red, increase, blue, decrease. **Right,** enriched pathways by DEGs analysis in Astrocytes from the NC and Prop 6h.

C. Left, Heat map shows differentially expressed genes in Astrocytes from the Sevo 6h and Sevo-R2h. Red, increase, blue, decrease. **Right**, enriched pathways by DEGs analysis in Astrocytes from the Sevo 6h and Sevo-R2h.

D. Left, Heat map shows differentially expressed genes in Astrocytes from the Prop 6h and Prop-R2h. Red, increase, blue, decrease. **Right**, enriched pathways by DEGs analysis in Astrocytes from the Prop 6h and Prop-R2h.

Interneurons

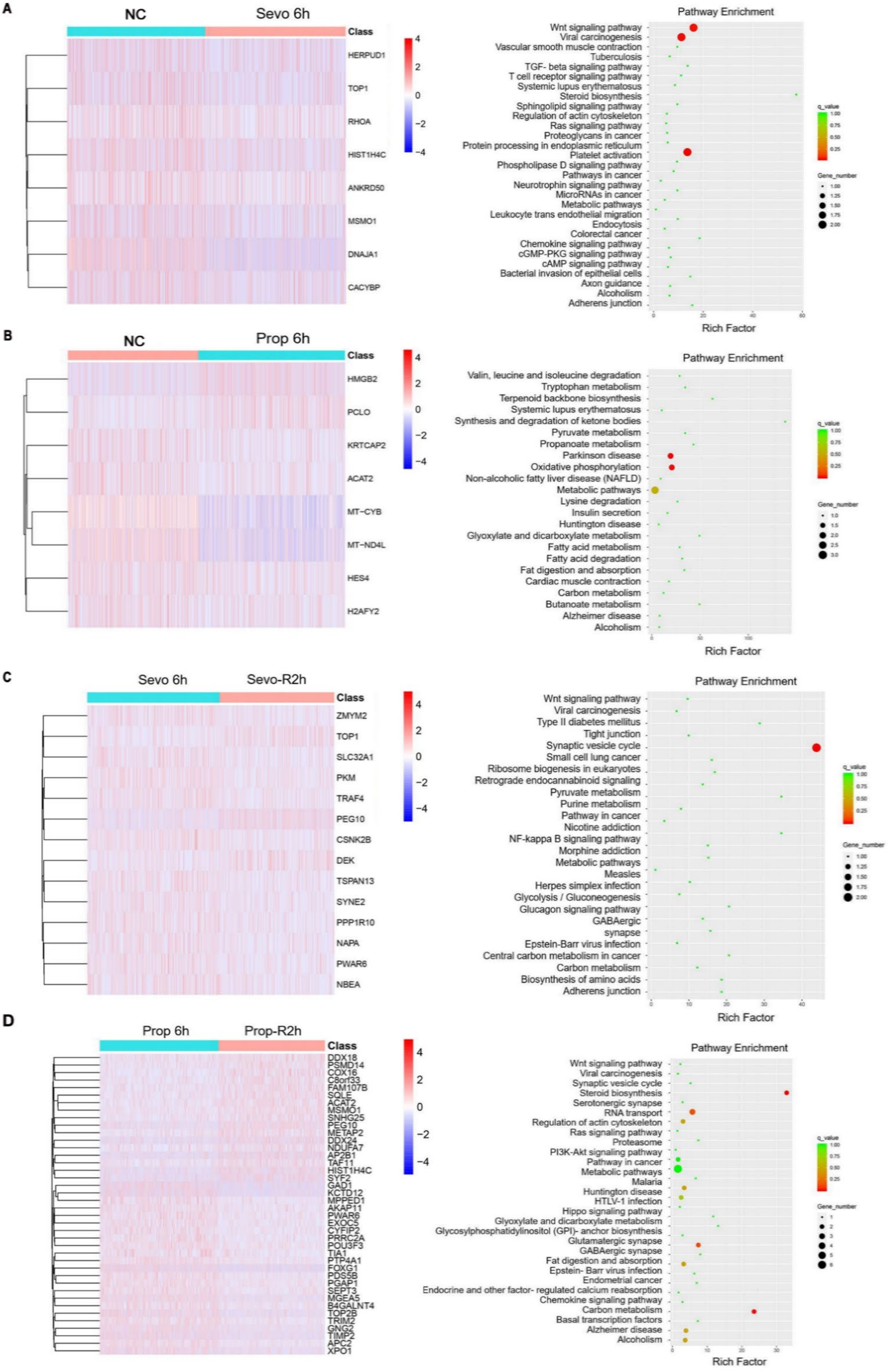

Figure S3. Differential expressed genes (DEGs) analysis and enriched pathways analysis of Interneurons, Related to Figure 3. Red, upregulation; blue, downregulation.
A. Left, Heat map shows differentially expressed genes in Interneurons from the NC and Sevo 6h. Red, increase, blue, decrease. **Right,** enriched pathways by DEGs analysis in Interneurons from the NC and Sevo 6h.
B. Left, Heat map shows differentially expressed genes in Interneurons from the NC and Prop 6h. Red, increase, blue, decrease. **Right,** enriched pathways by DEGs analysis in Interneurons from the NC and Prop 6h.
C. Left, Heat map shows differentially expressed genes in Interneurons from the Sevo 6h and Sevo-R2h. Red, increase, blue, decrease. **Right,** enriched pathways by DEGs analysis in Interneurons from the Sevo 6h and Sevo-R2h.
D. Left, Heat map shows differentially expressed genes in Interneurons from the Prop 6h and Prop-R2h. Red, increase, blue, decrease. **Right,** enriched pathways by DEGs analysis in Interneurons from the Prop 6h and Prop-R2h.

Microglia

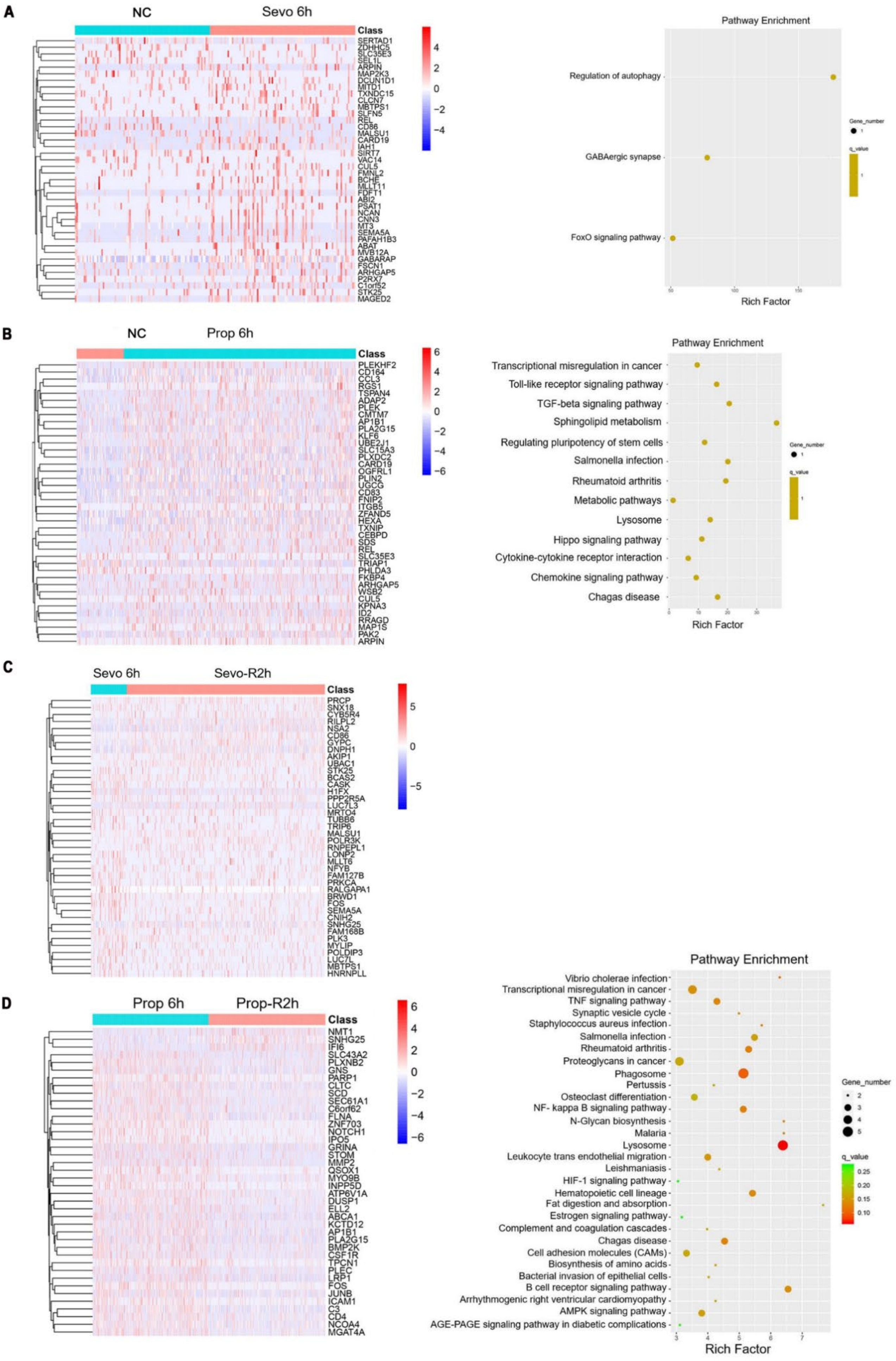
A
NC
Sevo 6h
Class
SERTAD1
ZDHHC5
SLC35E3
SEL1L
ARPIN
MAP2K3
DCUN1D1
MITD1
TXNDC15
CLCN7
MBTPS1
SLFN5
REL
CD86
MALSU1
CARD19
IAH1
SIRT7
VAC14
CUL5
FMNL2
BCHE
MLLT11
FDFT1
ABI2
PSAT1
NCAN
CNN3
MT3
SEMA5A
PAFAH1B3
ABAT
MVB12A
GABARAP
FSCN1
ARHGAP5
P2RX7
C1orf52
STK25
MAGED2
4
2
0
−2
−4
Pathway Enrichment
Regulation of autophagy
GABAergic synapse
FoxO signaling pathway
Rich Factor
Gene_number
q_value
B
NC
Prop 6h
Class
PLEKHF2
CD164
CCL3
RGS1
TSPAN4
ADAP2
PLEK
CMTM7
AP1B1
PLA2G15
KLF6
UBE2J1
SLC15A3
PLXDC2
CARD19
OGFRL1
PLIN2
UGCG
CD83
FNIP2
ITGB5
ZFAND5
HEXA
TXNIP
CEBPD
SDS
REL
SLC35E3
TRIAP1
PHLDA3
FKBP4
ARHGAP5
WSB2
CUL5
KPNA3
ID2
RRAGD
MAP1S
PAK2
ARPIN
6
4
2
0
−2
−4
−6
Pathway Enrichment
Transcriptional misregulation in cancer
Toll-like receptor signaling pathway
TGF-beta signaling pathway
Sphingolipid metabolism
Regulating pluripotency of stem cells
Salmonella infection
Rheumatoid arthritis
Metabolic pathways
Lysosome
Hippo signaling pathway
Cytokine-cytokine receptor interaction
Chemokine signaling pathway
Chagas disease
Rich Factor
Gene_number
q_value
C
Sevo 6h
Sevo-R2h
Class
PRCP
SNX18
CYB5R4
RILPL2
NSA2
CD86
GYPC
DNPH1
AKIP1
UBAC1
STK25
BCAS2
CASK
H1FX
PPP2R5A
LUC7L3
MRTO4
TUBB6
TRIP6
MALSU1
POLR3K
RNPEPL1
LONP2
MLLT6
NFYB
FAM127B
PRKCA
RALGAPA1
BRWD1
FOS
SEMA5A
CNIH2
SNHG25
FAM168B
PLK3
MYLIP
POLDIP3
LUC7L
MBTPS1
HNRNPLL
5
0
−5
D
Prop 6h
Prop-R2h
Class
NMT1
SNHG25
IFI6
SLC43A2
PLXNB2
GNS
PARP1
CLTC
SCD
SEC61A1
C6orf62
FLNA
ZNF703
NOTCH1
IPO5
GRINA
STOM
MMP2
QSOX1
MYO9B
INPP5D
ATP6V1A
DUSP1
ELL2
ABCA1
KCTD12
AP1B1
PLA2G15
BMP2K
CSF1R
TPCN1
PLEC
LRP1
FOS
JUNB
ICAM1
C3
CD4
NCOA4
MGAT4A
6
4
2
0
−2
−4
−6
Pathway Enrichment
Vibrio cholerae infection
Transcriptional misregulation in cancer
TNF signaling pathway
Synaptic vesicle cycle
Staphylococcus aureus infection
Salmonella infection
Rheumatoid arthritis
Proteoglycans in cancer
Phagosome
Pertussis
Osteoclast differentiation
NF- kappa B signaling pathway
N-Glycan biosynthesis
Malaria
Lysosome
Leukocyte trans endothelial migration
Leishmaniasis
HIF-1 signaling pathway
Hematopoietic cell lineage
Fat digestion and absorption
Estrogen signaling pathway
Complement and coagulation cascades
Chagas disease
Cell adhesion molecules (CAMs)
Biosynthesis of amino acids
Bacterial invasion of epithelial cells
B cell receptor signaling pathway
Arrhythmogenic right ventricular cardiomyopathy
AMPK signaling pathway
AGE-PAGE signaling pathway in diabetic complications
3
4
5
6
7
Rich Factor
Gene_number
2
3
4
5
q_value
0.25
0.20
0.15
0.10

Figure S4. Differential expressed genes (DEGs) and enriched pathways analysis of Microglia, Related to Figure 3. Red, upregulation; blue, downregulation.

A. Left, Heat map shows differentially expressed genes in Microglia from the NC and Sevo 6h. Red, increase, blue, decrease. **Right**, enriched pathways by DEGs analysis in Microglia from the NC and Sevo 6h.

B. Left, Heat map shows differentially expressed genes in Microglia from the NC and Prop 6h. Red, increase, blue, decrease. **Right,** enriched pathways by DEGs analysis in Microglia from the NC and Prop 6h.

C. Left, Heat map shows differentially expressed genes in Microglia from the Sevo 6h and Sevo-R2h. Red, increase, blue, decrease. **Right**, enriched pathways by DEGs analysis in Microglia from the Sevo 6h and Sevo-R2h.

D. Left, Heat map shows differentially expressed genes in Microglia from the Prop 6h and Prop-R2h. Red, increase, blue, decrease. **Right**, enriched pathways by DEGs analysis in Microglia from the Prop 6h and Prop-R2h.

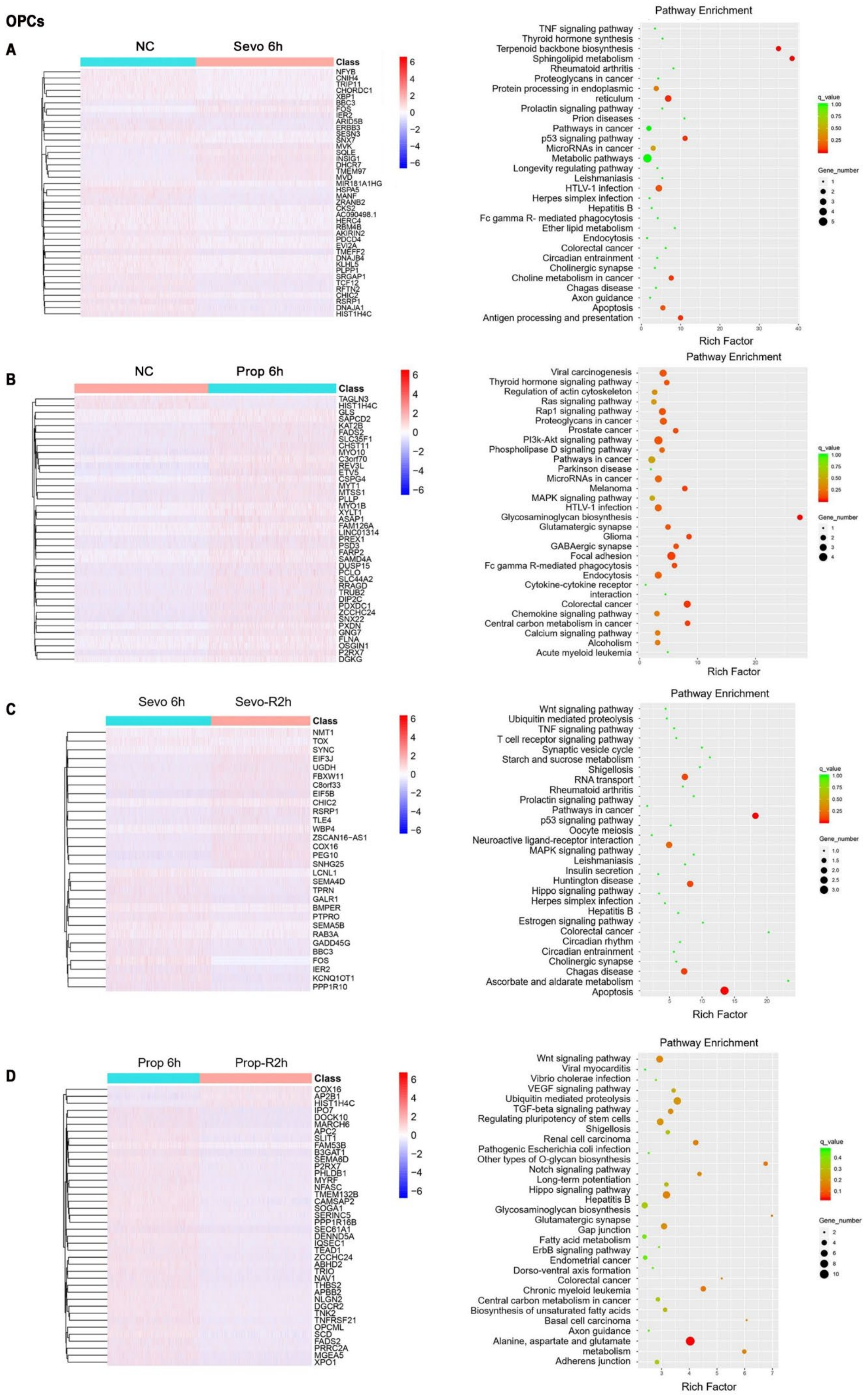

OPCs
A
NC
Sevo 6h
Class
NFYB
CNIH4
TRIP11
CHORDC1
XBP1
BBC3
FOS
IER2
ARID5B
ERBB3
SESN3
SNX7
MVK
SQLE
INSIG1
DHCR7
TMEM97
MVD
MIR181A1HG
HSPA5
MANF
ZRANB2
CKS2
AC090498.1
HERC4
RBM4B
AKIRIN2
PDCD4
EVI2A
TMEFF2
DNAJB4
KLHL5
PLPP1
SRGAP1
TCF12
RFTN2
CHIC2
RSRP1
DNAJA1
HIST1H4C
Pathway Enrichment
TNF signaling pathway
Thyroid hormone synthesis
Terpenoid backbone biosynthesis
Sphingolipid metabolism
Rheumatoid arthritis
Proteoglycans in cancer
Protein processing in endoplasmic reticulum
Prolactin signaling pathway
Prion diseases
Pathways in cancer
p53 signaling pathway
MicroRNAs in cancer
Metabolic pathways
Longevity regulating pathway
Leishmaniasis
HTLV-1 infection
Herpes simplex infection
Hepatitis B
Fc gamma R- mediated phagocytosis
Ether lipid metabolism
Endocytosis
Colorectal cancer
Circadian entrainment
Cholinergic synapse
Choline metabolism in cancer
Chagas disease
Axon guidance
Apoptosis
Antigen processing and presentation
Rich Factor
q_value
Gene_number
B
NC
Prop 6h
Class
TAGLN3
HIST1H4C
GLS
SAPCD2
KAT2B
FADS2
SLC35F1
CHST11
MYO10
C3orf70
REV3L
ETV5
CSPG4
MYT1
MTSS1
PLLP
MYO1B
XYLT1
ASAP1
FAM126A
LINC01314
PREX1
PSD3
FARP2
SAMD4A
DUSP15
PCLO
SLC44A2
RRAGD
TRUB2
DIP2C
PDXDC1
ZCCHC24
SNX22
PXDN
GNG7
FLNA
OSGIN1
P2RX7
DGKG
Pathway Enrichment
Viral carcinogenesis
Thyroid hormone signaling pathway
Regulation of actin cytoskeleton
Ras signaling pathway
Rap1 signaling pathway
Proteoglycans in cancer
Prostate cancer
PI3k-Akt signaling pathway
Phospholipase D signaling pathway
Pathways in cancer
Parkinson disease
MicroRNAs in cancer
Melanoma
MAPK signaling pathway
HTLV-1 infection
Glycosaminoglycan biosynthesis
Glutamatergic synapse
Glioma
GABAergic synapse
Focal adhesion
Fc gamma R-mediated phagocytosis
Endocytosis
Cytokine-cytokine receptor interaction
Colorectal cancer
Chemokine signaling pathway
Central carbon metabolism in cancer
Calcium signaling pathway
Alcoholism
Acute myeloid leukemia
Rich Factor
q_value
Gene_number
C
Sevo 6h
Sevo-R2h
Class
NMT1
TOX
SYNC
EIF3J
UGDH
FBXW11
C8orf33
EIF5B
CHIC2
RSRP1
TLE4
WBP4
ZSCAN16-AS1
COX16
PEG10
SNHG25
LCNL1
SEMA4D
TPRN
GALR1
BMPER
PTPRO
SEMA5B
RAB3A
GADD45G
BBC3
FOS
IER2
KCNQ1OT1
PPP1R10
Pathway Enrichment
Wnt signaling pathway
Ubiquitin mediated proteolysis
TNF signaling pathway
T cell receptor signaling pathway
Synaptic vesicle cycle
Starch and sucrose metabolism
Shigellosis
RNA transport
Rheumatoid arthritis
Prolactin signaling pathway
Pathways in cancer
p53 signaling pathway
Oocyte meiosis
Neuroactive ligand-receptor interaction
MAPK signaling pathway
Leishmaniasis
Insulin secretion
Huntington disease
Hippo signaling pathway
Herpes simplex infection
Hepatitis B
Estrogen signaling pathway
Colorectal cancer
Circadian rhythm
Circadian entrainment
Cholinergic synapse
Chagas disease
Ascorbate and aldarate metabolism
Apoptosis
Rich Factor
q_value
Gene_number
D
Prop 6h
Prop-R2h
Class
COX16
AP2B1
HIST1H4C
IPO7
DOCK10
MARCH6
APC2
SLIT1
FAM53B
B3GAT1
SEMA6D
P2RX7
PHLDB1
MYRF
NFASC
TMEM132B
CAMSAP2
SOGA1
SERINC5
PPP1R16B
SEC61A1
DENND5A
IQSEC1
TEAD1
ZCCHC24
ABHD2
TRIO
NAV1
THBS2
APBB2
NLGN2
DGCR2
TNK2
TNFRSF21
OPCML
SCD
FADS2
PRRC2A
MGEA5
XPO1
Pathway Enrichment
Wnt signaling pathway
Viral myocarditis
Vibrio cholerae infection
VEGF signaling pathway
Ubiquitin mediated proteolysis
TGF-beta signaling pathway
Regulating pluripotency of stem cells
Shigellosis
Renal cell carcinoma
Pathogenic Escherichia coli infection
Other types of O-glycan biosynthesis
Notch signaling pathway
Long-term potentiation
Hippo signaling pathway
Hepatitis B
Glycosaminoglycan biosynthesis
Glutamatergic synapse
Gap junction
Fatty acid metabolism
ErbB signaling pathway
Endometrial cancer
Dorso-ventral axis formation
Colorectal cancer
Chronic myeloid leukemia
Central carbon metabolism in cancer
Biosynthesis of unsaturated fatty acids
Basal cell carcinoma
Axon guidance
Alanine, aspartate and glutamate metabolism
Adherens junction
Rich Factor
q_value
Gene_number

Figure S5. Differential expressed genes (DEGs) and enriched pathways analysis of OPCs, Related to Figure 3. Red, upregulation; blue, downregulation.

A. Left, Heat map shows differentially expressed genes in OPCs from the NC and Sevo 6h. Red, increase, blue, decrease. **Right**, enriched pathways by DEGs analysis in OPCs from the NC and Sevo 6h.

B. Left, Heat map shows differentially expressed genes in OPCs from the NC and Prop 6h. Red, increase, blue, decrease. **Right**, enriched pathways by DEGs analysis in OPCs from the NC and Prop 6h.

C. Left, Heat map shows differentially expressed genes in OPCs from the Sevo 6h and Sevo-R2h. Red, increase, blue, decrease. **Right**, enriched pathways by DEGs analysis in OPCs from the Sevo 6h and Sevo-R2h.

D. Left, Heat map shows differentially expressed genes in OPCs from the Prop 6h and Prop-R2h. Red, increase, blue, decrease. **Right**, enriched pathways by DEGs analysis in OPCs from the Prop 6h and Prop-R2h.

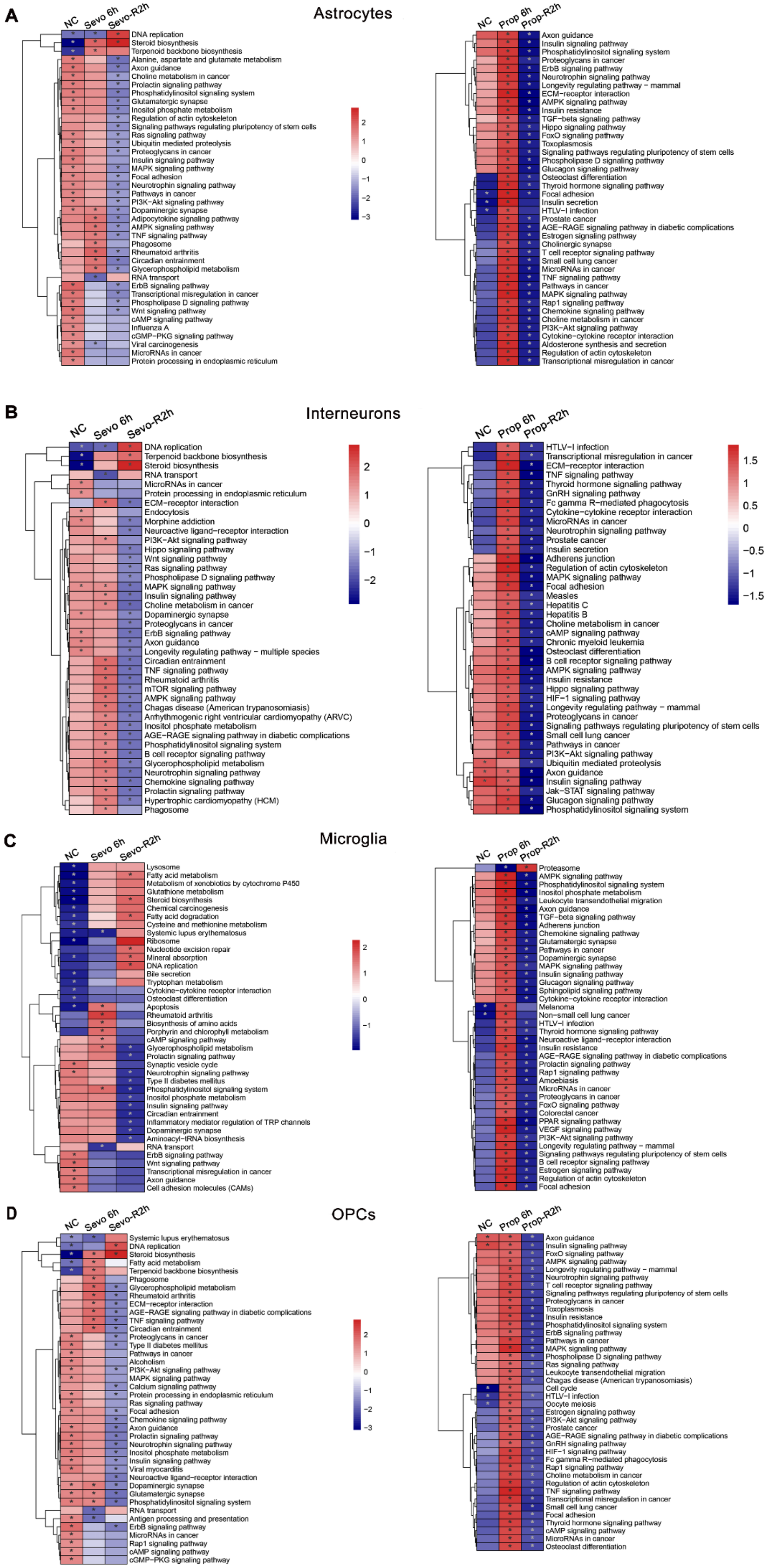

A
Astrocytes
NC
Sevo 6h
Sevo-R2h
DNA replication
Steroid biosynthesis
Terpenoid backbone biosynthesis
Alanine, aspartate and glutamate metabolism
Axon guidance
Choline metabolism in cancer
Prolactin signaling pathway
Phosphatidylinositol signaling system
Glutamatergic synapse
Inositol phosphate metabolism
Regulation of actin cytoskeleton
Signaling pathways regulating pluripotency of stem cells
Ras signaling pathway
Ubiquitin mediated proteolysis
Proteoglycans in cancer
Insulin signaling pathway
MAPK signaling pathway
Focal adhesion
Neurotrophin signaling pathway
Pathways in cancer
PI3K−Akt signaling pathway
Dopaminergic synapse
Adipocytokine signaling pathway
AMPK signaling pathway
TNF signaling pathway
Phagosome
Rheumatoid arthritis
Circadian entrainment
Glycerophospholipid metabolism
RNA transport
ErbB signaling pathway
Transcriptional misregulation in cancer
Phospholipase D signaling pathway
Wnt signaling pathway
cAMP signaling pathway
Influenza A
cGMP−PKG signaling pathway
Viral carcinogenesis
MicroRNAs in cancer
Protein processing in endoplasmic reticulum
2
1
0
−1
−2
−3
NC
Prop 6h
Prop-R2h
Axon guidance
Insulin signaling pathway
Phosphatidylinositol signaling system
Proteoglycans in cancer
ErbB signaling pathway
Neurotrophin signaling pathway
Longevity regulating pathway − mammal
ECM−receptor interaction
AMPK signaling pathway
Insulin resistance
TGF−beta signaling pathway
Hippo signaling pathway
FoxO signaling pathway
Toxoplasmosis
Signaling pathways regulating pluripotency of stem cells
Phospholipase D signaling pathway
Glucagon signaling pathway
Osteoclast differentiation
Thyroid hormone signaling pathway
Focal adhesion
Insulin secretion
HTLV−I infection
Prostate cancer
AGE−RAGE signaling pathway in diabetic complications
Estrogen signaling pathway
Cholinergic synapse
T cell receptor signaling pathway
Small cell lung cancer
MicroRNAs in cancer
TNF signaling pathway
Pathways in cancer
MAPK signaling pathway
Rap1 signaling pathway
Chemokine signaling pathway
Choline metabolism in cancer
PI3K−Akt signaling pathway
Cytokine−cytokine receptor interaction
Aldosterone synthesis and secretion
Regulation of actin cytoskeleton
Transcriptional misregulation in cancer
1.5
1
0.5
0
−0.5
−1
−1.5
B
Interneurons
NC
Sevo 6h
Sevo-R2h
DNA replication
Terpenoid backbone biosynthesis
Steroid biosynthesis
RNA transport
MicroRNAs in cancer
Protein processing in endoplasmic reticulum
ECM−receptor interaction
Endocytosis
Morphine addiction
Neuroactive ligand−receptor interaction
PI3K−Akt signaling pathway
Hippo signaling pathway
Wnt signaling pathway
Ras signaling pathway
Phospholipase D signaling pathway
MAPK signaling pathway
Insulin signaling pathway
Choline metabolism in cancer
Dopaminergic synapse
Proteoglycans in cancer
ErbB signaling pathway
Axon guidance
Longevity regulating pathway − multiple species
Circadian entrainment
TNF signaling pathway
Rheumatoid arthritis
mTOR signaling pathway
AMPK signaling pathway
Chagas disease (American trypanosomiasis)
Arrhythmogenic right ventricular cardiomyopathy (ARVC)
Inositol phosphate metabolism
AGE−RAGE signaling pathway in diabetic complications
Phosphatidylinositol signaling system
B cell receptor signaling pathway
Glycerophospholipid metabolism
Neurotrophin signaling pathway
Chemokine signaling pathway
Prolactin signaling pathway
Hypertrophic cardiomyopathy (HCM)
Phagosome
2
1
0
−1
−2
NC
Prop 6h
Prop-R2h
HTLV−I infection
Transcriptional misregulation in cancer
ECM−receptor interaction
TNF signaling pathway
Thyroid hormone signaling pathway
GnRH signaling pathway
Fc gamma R−mediated phagocytosis
Cytokine−cytokine receptor interaction
MicroRNAs in cancer
Neurotrophin signaling pathway
Prostate cancer
Insulin secretion
Adherens junction
Regulation of actin cytoskeleton
MAPK signaling pathway
Focal adhesion
Measles
Hepatitis C
Hepatitis B
Choline metabolism in cancer
cAMP signaling pathway
Chronic myeloid leukemia
Osteoclast differentiation
B cell receptor signaling pathway
AMPK signaling pathway
Insulin resistance
Hippo signaling pathway
HIF−1 signaling pathway
Longevity regulating pathway − mammal
Proteoglycans in cancer
Signaling pathways regulating pluripotency of stem cells
Small cell lung cancer
Pathways in cancer
PI3K−Akt signaling pathway
Ubiquitin mediated proteolysis
Axon guidance
Insulin signaling pathway
Jak−STAT signaling pathway
Glucagon signaling pathway
Phosphatidylinositol signaling system
1.5
1
0.5
0
−0.5
−1
−1.5
C
Microglia
NC
Sevo 6h
Sevo-R2h
Lysosome
Fatty acid metabolism
Metabolism of xenobiotics by cytochrome P450
Glutathione metabolism
Steroid biosynthesis
Chemical carcinogenesis
Fatty acid degradation
Cysteine and methionine metabolism
Systemic lupus erythematosus
Ribosome
Nucleotide excision repair
Mineral absorption
DNA replication
Bile secretion
Tryptophan metabolism
Cytokine−cytokine receptor interaction
Osteoclast differentiation
Apoptosis
Rheumatoid arthritis
Biosynthesis of amino acids
Porphyrin and chlorophyll metabolism
cAMP signaling pathway
Glycerophospholipid metabolism
Prolactin signaling pathway
Synaptic vesicle cycle
Neurotrophin signaling pathway
Type II diabetes mellitus
Phosphatidylinositol signaling system
Inositol phosphate metabolism
Insulin signaling pathway
Circadian entrainment
Inflammatory mediator regulation of TRP channels
Dopaminergic synapse
Aminoacyl−tRNA biosynthesis
RNA transport
ErbB signaling pathway
Wnt signaling pathway
Transcriptional misregulation in cancer
Axon guidance
Cell adhesion molecules (CAMs)
2
1
0
−1
NC
Prop 6h
Prop-R2h
Proteasome
AMPK signaling pathway
Phosphatidylinositol signaling system
Inositol phosphate metabolism
Leukocyte transendothelial migration
Axon guidance
TGF−beta signaling pathway
Adherens junction
Chemokine signaling pathway
Glutamatergic synapse
Pathways in cancer
Dopaminergic synapse
MAPK signaling pathway
Insulin signaling pathway
Glucagon signaling pathway
Sphingolipid signaling pathway
Cytokine−cytokine receptor interaction
Melanoma
Non−small cell lung cancer
HTLV−I infection
Thyroid hormone signaling pathway
Neuroactive ligand−receptor interaction
Insulin resistance
AGE−RAGE signaling pathway in diabetic complications
Prolactin signaling pathway
Rap1 signaling pathway
Amoebiasis
MicroRNAs in cancer
Proteoglycans in cancer
FoxO signaling pathway
Colorectal cancer
PPAR signaling pathway
VEGF signaling pathway
PI3K−Akt signaling pathway
Longevity regulating pathway − mammal
Signaling pathways regulating pluripotency of stem cells
B cell receptor signaling pathway
Estrogen signaling pathway
Regulation of actin cytoskeleton
Focal adhesion
1.5
1
0.5
0
−0.5
−1
−1.5
D
OPCs
NC
Sevo 6h
Sevo-R2h
Systemic lupus erythematosus
DNA replication
Steroid biosynthesis
Fatty acid metabolism
Terpenoid backbone biosynthesis
Phagosome
Glycerophospholipid metabolism
Rheumatoid arthritis
ECM−receptor interaction
AGE−RAGE signaling pathway in diabetic complications
TNF signaling pathway
Circadian entrainment
Proteoglycans in cancer
Type II diabetes mellitus
Pathways in cancer
Alcoholism
PI3K−Akt signaling pathway
MAPK signaling pathway
Calcium signaling pathway
Protein processing in endoplasmic reticulum
Ras signaling pathway
Focal adhesion
Chemokine signaling pathway
Axon guidance
Prolactin signaling pathway
Neurotrophin signaling pathway
Inositol phosphate metabolism
Insulin signaling pathway
Viral myocarditis
Neuroactive ligand−receptor interaction
Dopaminergic synapse
Glutamatergic synapse
Phosphatidylinositol signaling system
RNA transport
Antigen processing and presentation
ErbB signaling pathway
MicroRNAs in cancer
Rap1 signaling pathway
cAMP signaling pathway
cGMP−PKG signaling pathway
2
1
0
−1
−2
−3
NC
Prop 6h
Prop-R2h
Axon guidance
Insulin signaling pathway
FoxO signaling pathway
AMPK signaling pathway
Longevity regulating pathway − mammal
Neurotrophin signaling pathway
T cell receptor signaling pathway
Signaling pathways regulating pluripotency of stem cells
Proteoglycans in cancer
Toxoplasmosis
Insulin resistance
Phosphatidylinositol signaling system
ErbB signaling pathway
Pathways in cancer
MAPK signaling pathway
Phospholipase D signaling pathway
Ras signaling pathway
Leukocyte transendothelial migration
Chagas disease (American trypanosomiasis)
Cell cycle
HTLV−I infection
Oocyte meiosis
Estrogen signaling pathway
PI3K−Akt signaling pathway
Prostate cancer
AGE−RAGE signaling pathway in diabetic complications
GnRH signaling pathway
HIF−1 signaling pathway
Fc gamma R−mediated phagocytosis
Rap1 signaling pathway
Choline metabolism in cancer
Regulation of actin cytoskeleton
TNF signaling pathway
Transcriptional misregulation in cancer
Small cell lung cancer
Focal adhesion
Thyroid hormone signaling pathway
cAMP signaling pathway
MicroRNAs in cancer
Osteoclast differentiation
1
0
−1
−2

Figure S6. Gene set enrichment analysis (GSEA) of Astrocytes, Interneurons, Microglia and OPCs treated with sevoflurane or propofol followed by recovery, Related to Figure 3.
A. Left, GSEA of Astrocytes from NC, Sevo 6h and Sevo-R2h. **Right**, GSEA of Astrocytes from NC, Prop 6h and Prop-R2h. Red, increase, blue, decrease. * $P < 0.05$.
B. Left, GSEA of Interneurons from NC, Sevo 6h and Sevo-R2h. **Right**, GSEA of interneurons from NC, Prop 6h and Prop-R2h. Red, increase, blue, decrease. * $P < 0.05$.
C. Left, GSEA of Microglia from NC, Sevo 6h and Sevo-R2h. **Right**, GSEA of Microglia from NC, Prop 6h and Prop-R2h. Red, increase, blue, decrease. * $P < 0.05$.
D. Left, GSEA of OPCs from NC, Sevo 6h and Sevo-R2h. **Right**, GSEA of OPCs from NC, Prop 6h and Prop-R2h. Red, increase, blue, decrease. * $P < 0.05$.

All

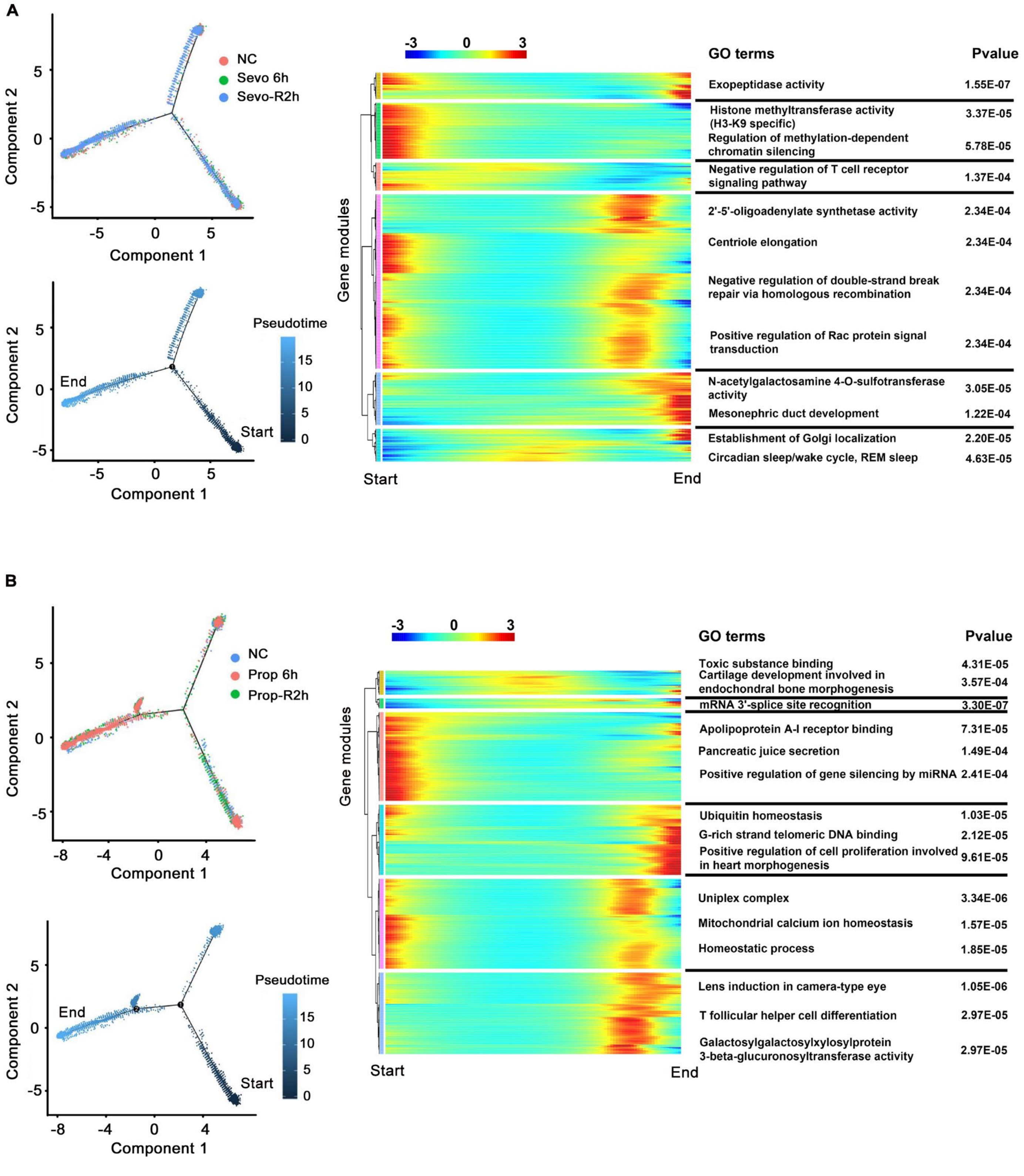

A
NC
Sevo 6h
Sevo-R2h
Component 2
Component 1
Pseudotime
End
Start
Gene modules
GO terms
Pvalue
Exopeptidase activity 1.55E-07
Histone methyltransferase activity (H3-K9 specific) 3.37E-05
Regulation of methylation-dependent chromatin silencing 5.78E-05
Negative regulation of T cell receptor signaling pathway 1.37E-04
2'-5'-oligoadenylate synthetase activity 2.34E-04
Centriole elongation 2.34E-04
Negative regulation of double-strand break repair via homologous recombination 2.34E-04
Positive regulation of Rac protein signal transduction 2.34E-04
N-acetylgalactosamine 4-O-sulfotransferase activity 3.05E-05
Mesonephric duct development 1.22E-04
Establishment of Golgi localization 2.20E-05
Circadian sleep/wake cycle, REM sleep 4.63E-05
B
NC
Prop 6h
Prop-R2h
Toxic substance binding 4.31E-05
Cartilage development involved in endochondral bone morphogenesis 3.57E-04
mRNA 3'-splice site recognition 3.30E-07
Apolipoprotein A-I receptor binding 7.31E-05
Pancreatic juice secretion 1.49E-04
Positive regulation of gene silencing by miRNA 2.41E-04
Ubiquitin homeostasis 1.03E-05
G-rich strand telomeric DNA binding 2.12E-05
Positive regulation of cell proliferation involved in heart morphogenesis 9.61E-05
Uniplex complex 3.34E-06
Mitochondrial calcium ion homeostasis 1.57E-05
Homeostatic process 1.85E-05
Lens induction in camera-type eye 1.05E-06
T follicular helper cell differentiation 2.97E-05
Galactosylgalactosylxylosylprotein 3-beta-glucuronosyltransferase activity 2.97E-05

Figure S7. Single-cell trajectory analysis of all clusters of cells treated with sevoflurane or propofol for 6h with 2h recovery, Related to Figure 4.

A. Left, upper, pseudo time series analysis results of all clusters of cells in NC, Sevo 6h and Sevo-R6h groups; lower, these three groups from start to end by monocle pseudo time series analysis. **Right**, the bifurcation of gene expression from start to end is clustered hierarchically into six modules. Gene ontology analysis of each module reflected the processes controlling all clusters of cells by sevoflurane. In this heatmap, columns are points in pseudo-time, rows are genes, and left represents the beginning of pseudo-time. The start point of heatmap is the same as the pseudo time series analysis. Red, upregulation; blue, downregulation.

B. Left, up, pseudo time series analysis results of all cluster cells from with group NC, Prop 6h and Prop-R6h, down, these three groups from start to end by monocle pseudo time series analysis. **Right,** the bifurcation of gene expression along from start to end is clustered hierarchically into six modules. Gene ontology analysis of each module reflected the processes controlling all cluster cells by propofol. In this heat map, columns are points in pseudo-time, rows are genes, and the left is the beginning of pseudo-time. The start point of the heat map is the same as the pseudo time series analysis. Red, upregulation; blue, downregulation.

Astrocytes

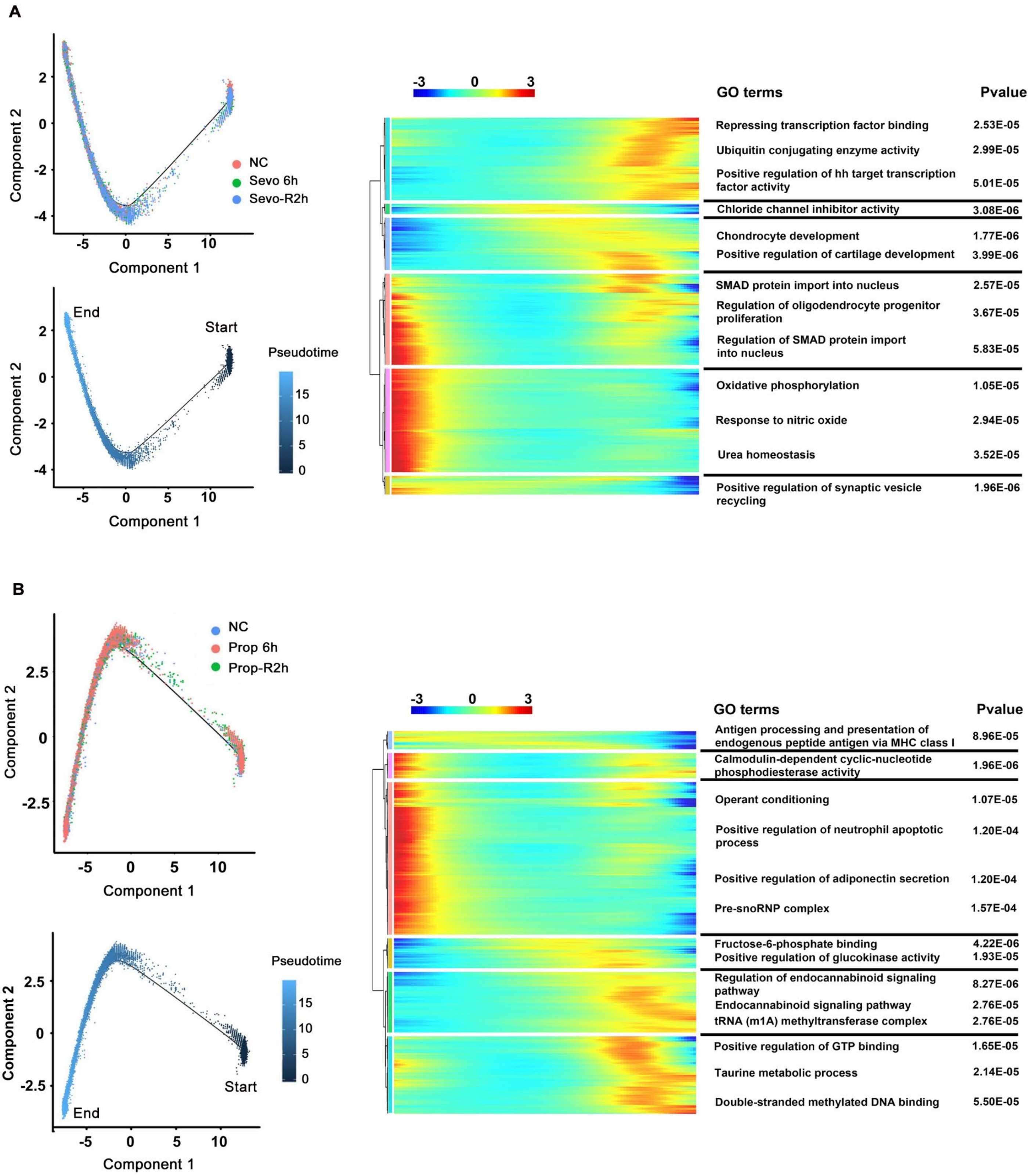
A
Component 2
Component 1
NC
Sevo 6h
Sevo-R2h
End
Start
Pseudotime
GO terms
Pvalue
Repressing transcription factor binding 2.53E-05
Ubiquitin conjugating enzyme activity 2.99E-05
Positive regulation of hh target transcription factor activity 5.01E-05
Chloride channel inhibitor activity 3.08E-06
Chondrocyte development 1.77E-06
Positive regulation of cartilage development 3.99E-06
SMAD protein import into nucleus 2.57E-05
Regulation of oligodendrocyte progenitor proliferation 3.67E-05
Regulation of SMAD protein import into nucleus 5.83E-05
Oxidative phosphorylation 1.05E-05
Response to nitric oxide 2.94E-05
Urea homeostasis 3.52E-05
Positive regulation of synaptic vesicle recycling 1.96E-06
B
NC
Prop 6h
Prop-R2h
Component 2
Component 1
Pseudotime
End
Start
GO terms
Pvalue
Antigen processing and presentation of endogenous peptide antigen via MHC class I 8.96E-05
Calmodulin-dependent cyclic-nucleotide phosphodiesterase activity 1.96E-06
Operant conditioning 1.07E-05
Positive regulation of neutrophil apoptotic process 1.20E-04
Positive regulation of adiponectin secretion 1.20E-04
Pre-snoRNP complex 1.57E-04
Fructose-6-phosphate binding 4.22E-06
Positive regulation of glucokinase activity 1.93E-05
Regulation of endocannabinoid signaling pathway 8.27E-06
Endocannabinoid signaling pathway 2.76E-05
tRNA (m1A) methyltransferase complex 2.76E-05
Positive regulation of GTP binding 1.65E-05
Taurine metabolic process 2.14E-05
Double-stranded methylated DNA binding 5.50E-05

Figure S8. Single-cell trajectory analysis of Astrocytes treated with sevoflurane and propofol for 6h and recover for 2h, Related to Figure 4.

A. Left, up, pseudo time series analysis results of Astrocytes from with group NC, Sevo 6h and Sevo-R6h, down, these three groups from start to end by monocle pseudo time series analysis. **Right,** the bifurcation of gene expression along from start to end is clustered hierarchically into six modules. Gene ontology analysis of each module reflected the processes controlling Astrocytes by sevoflurane. In this heat map, columns are points in pseudo-time, rows are genes, and the left is the beginning of pseudo-time. The start point of the heat map is the same as the pseudo time series analysis. Red, upregulation; blue, downregulation.

B. Left, up, pseudo time series analysis results of Astrocytes from with group NC, Prop 6h and Prop-R6h, down, these three groups from start to end by monocle pseudo time series analysis. **Right**, the bifurcation of gene expression along from start to end is clustered hierarchically into six modules. Gene ontology analysis of each module reflected the processes controlling astrocytes by propofol. In this heat map, columns are points in pseudo-time, rows are genes, and the left is the beginning of pseudo-time. The start point of the heat map is the same as the pseudo time series analysis. Red, upregulation; blue, downregulation.

OPCs

A

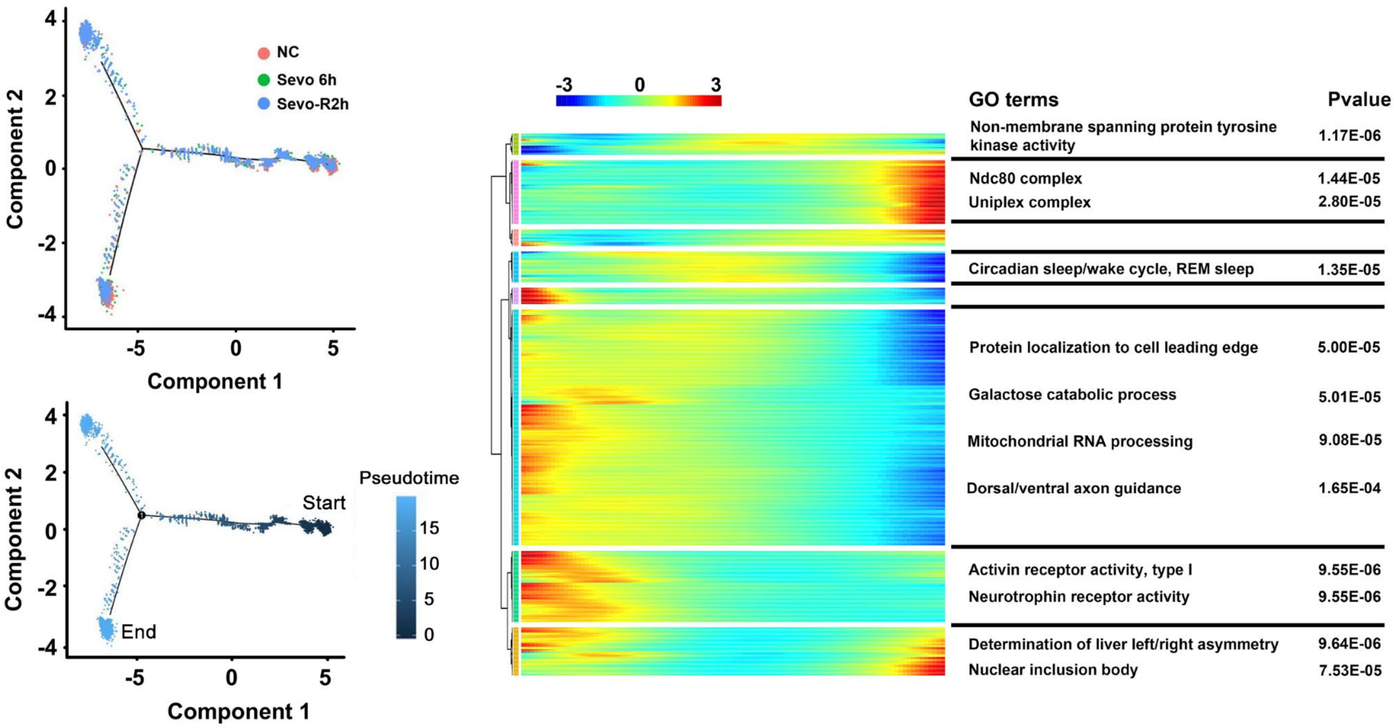
NC
Sevo 6h
Sevo-R2h
Component 2
Component 1
Pseudotime
Start
End
-3 0 3
GO terms Pvalue
Non-membrane spanning protein tyrosine kinase activity 1.17E-06
Ndc80 complex 1.44E-05
Uniplex complex 2.80E-05
Circadian sleep/wake cycle, REM sleep 1.35E-05
Protein localization to cell leading edge 5.00E-05
Galactose catabolic process 5.01E-05
Mitochondrial RNA processing 9.08E-05
Dorsal/ventral axon guidance 1.65E-04
Activin receptor activity, type I 9.55E-06
Neurotrophin receptor activity 9.55E-06
Determination of liver left/right asymmetry 9.64E-06
Nuclear inclusion body 7.53E-05

B

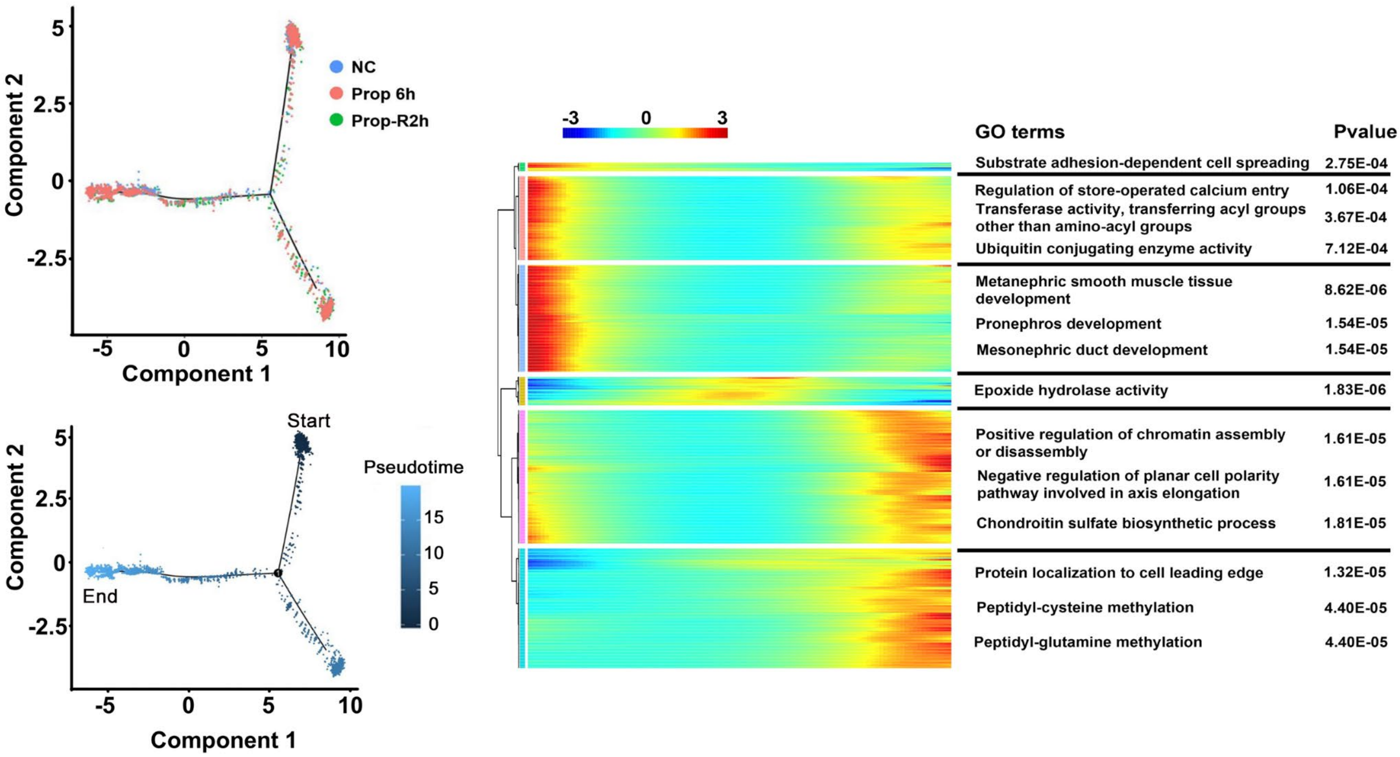
NC
Prop 6h
Prop-R2h
Component 2
Component 1
Pseudotime
Start
End
-3 0 3
GO terms Pvalue
Substrate adhesion-dependent cell spreading 2.75E-04
Regulation of store-operated calcium entry 1.06E-04
Transferase activity, transferring acyl groups other than amino-acyl groups 3.67E-04
Ubiquitin conjugating enzyme activity 7.12E-04
Metanephric smooth muscle tissue development 8.62E-06
Pronephros development 1.54E-05
Mesonephric duct development 1.54E-05
Epoxide hydrolase activity 1.83E-06
Positive regulation of chromatin assembly or disassembly 1.61E-05
Negative regulation of planar cell polarity pathway involved in axis elongation 1.61E-05
Chondroitin sulfate biosynthetic process 1.81E-05
Protein localization to cell leading edge 1.32E-05
Peptidyl-cysteine methylation 4.40E-05
Peptidyl-glutamine methylation 4.40E-05

Figure S9. Single-cell trajectory analysis of OPCs treated with sevoflurane and propofol for 6h and recover for 2h, Related to Figure 4.

A. Left, up, pseudo time series analysis results of OPCs from with group NC, Sevo 6h and Sevo-R6h, down, these three groups from start to end by monocle pseudo time series analysis. **Right,** the bifurcation of gene expression along from start to end is clustered hierarchically into six modules. Gene ontology analysis of each module reflected the processes controlling OPCs by sevoflurane. In this heat map, columns are points in pseudo-time, rows are genes, and the left is the beginning of pseudo-time. The start point of the heat map is the same as the pseudo time series analysis. Red, upregulation; blue, downregulation.

B. Left, up, pseudo time series analysis results of OPCs from with group NC, Prop 6h and Prop-R6h, down, these three groups from start to end by monocle pseudo time series analysis. **Right**, the bifurcation of gene expression along from start to end is clustered hierarchically into six modules. Gene ontology analysis of each module reflected the processes controlling OPCs by propofol. In this heat map, columns are points in pseudo-time, rows are genes, and the left is the beginning of pseudo-time. The start point of the heat map is the same as the pseudo time series analysis. Red, upregulation; blue, downregulation.

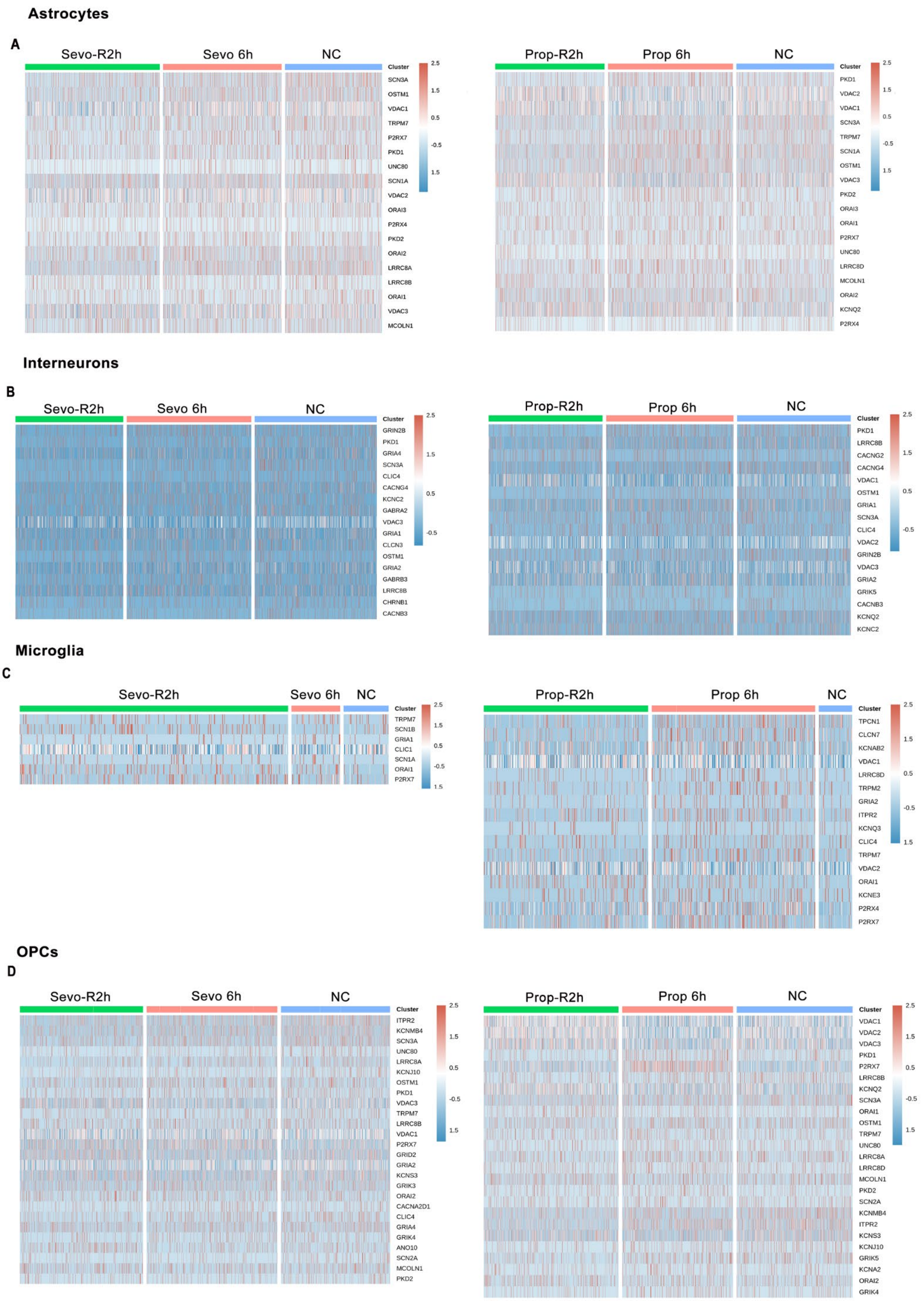
Astrocytes
A
Sevo-R2h
Sevo 6h
NC
Cluster
SCN3A
OSTM1
VDAC1
TRPM7
P2RX7
PKD1
UNC80
SCN1A
VDAC2
ORAI3
P2RX4
PKD2
ORAI2
LRRC8A
LRRC8B
ORAI1
VDAC3
MCOLN1
Prop-R2h
Prop 6h
NC
Cluster
PKD1
VDAC2
VDAC1
SCN3A
TRPM7
SCN1A
OSTM1
VDAC3
PKD2
ORAI3
ORAI1
P2RX7
UNC80
LRRC8D
MCOLN1
ORAI2
KCNQ2
P2RX4
Interneurons
B
Sevo-R2h
Sevo 6h
NC
Cluster
GRIN2B
PKD1
GRIA4
SCN3A
CLIC4
CACNG4
KCNC2
GABRA2
VDAC3
GRIA1
CLCN3
OSTM1
GRIA2
GABRB3
LRRC8B
CHRNB1
CACNB3
Prop-R2h
Prop 6h
NC
Cluster
PKD1
LRRC8B
CACNG2
CACNG4
VDAC1
OSTM1
GRIA1
SCN3A
CLIC4
VDAC2
GRIN2B
VDAC3
GRIA2
GRIK5
CACNB3
KCNQ2
KCNC2
Microglia
C
Sevo-R2h
Sevo 6h
NC
Cluster
TRPM7
SCN1B
GRIA1
CLIC1
SCN1A
ORAI1
P2RX7
Prop-R2h
Prop 6h
NC
Cluster
TPCN1
CLCN7
KCNAB2
VDAC1
LRRC8D
TRPM2
GRIA2
ITPR2
KCNQ3
CLIC4
TRPM7
VDAC2
ORAI1
KCNE3
P2RX4
P2RX7
OPCs
D
Sevo-R2h
Sevo 6h
NC
Cluster
ITPR2
KCNMB4
SCN3A
UNC80
LRRC8A
KCNJ10
OSTM1
PKD1
VDAC3
TRPM7
LRRC8B
VDAC1
P2RX7
GRID2
GRIA2
KCNS3
GRIK3
ORAI2
CACNA2D1
CLIC4
GRIA4
GRIK4
ANO10
SCN2A
MCOLN1
PKD2
Prop-R2h
Prop 6h
NC
Cluster
VDAC1
VDAC2
VDAC3
PKD1
P2RX7
LRRC8B
KCNQ2
SCN3A
ORAI1
OSTM1
TRPM7
UNC80
LRRC8A
LRRC8D
MCOLN1
PKD2
SCN2A
KCNMB4
ITPR2
KCNS3
KCNJ10
GRIK5
KCNA2
ORAI2
GRIK4
2.5
1.5
0.5
-0.5
1.5

Figure S10. Heatmap showing cell membrane ion channel gene differences of Astrocytes, interneurons, Microglia and OPCs treated by sevoflurane or propofol, Related to Figure 5. **A, B, C, D, E, F. Left,** the differences of ion channel genes in Astrocytes, interneurons, Microglia and OPCs treated with sevoflurane 6h and recover-2h were compared. **Right,** the differences of ion channel genes in astrocytes, interneurons, Microglia and OPCs treated with propofol 6h and recover-2h were compared. Red, increase, blue, decrease.

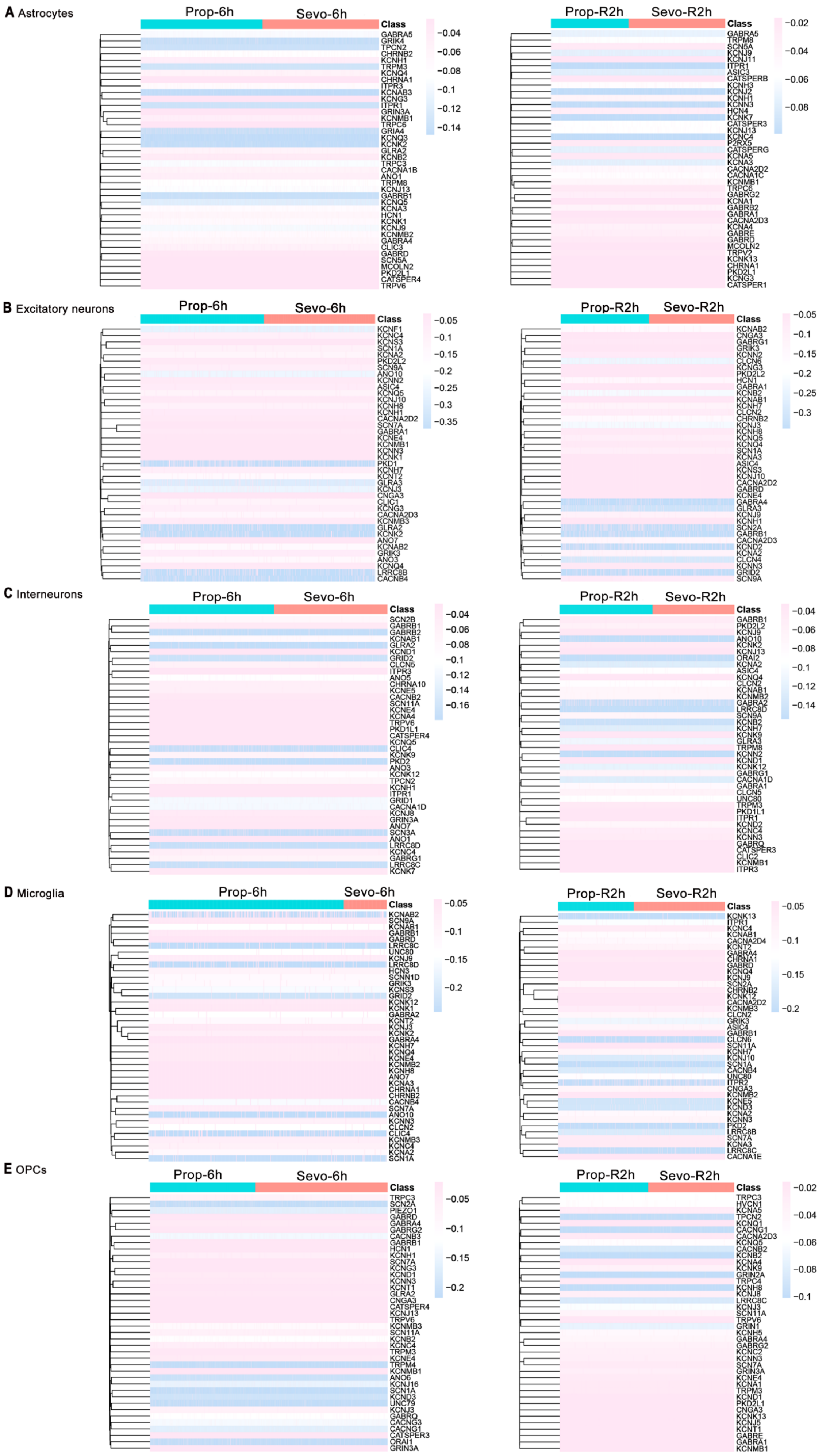
A Astrocytes
Prop-6h
Sevo-6h
Class
GABRA5
GRIK4
TPCN2
CHRNB2
KCNH1
TRPM3
KCNQ4
CHRNA1
ITPR3
KCNAB3
KCNG3
ITPR1
GRIN3A
KCNMB1
TRPC6
GRIA4
KCNQ3
KCNK2
GLRA2
KCNB2
TRPC3
CACNA1B
ANO1
TRPM8
KCNJ13
GABRB1
KCNQ5
KCNA3
HCN1
KCNK1
KCNJ9
KCNMB2
GABRA4
CLIC3
GABRD
SCN5A
MCOLN2
PKD2L1
CATSPER4
TRPV6
−0.04
−0.06
−0.08
−0.1
−0.12
−0.14
Prop-R2h
Sevo-R2h
Class
GABRA5
TRPM8
SCN5A
KCNJ9
KCNJ11
ITPR1
ASIC3
CATSPERB
KCNH3
KCNJ2
KCNH1
KCNN3
HCN4
KCNK7
CATSPER3
KCNJ13
KCNC4
P2RX5
CATSPERG
KCNA5
KCNA3
CACNA2D2
CACNA1C
KCNMB1
TRPC6
GABRG2
KCNA1
GABRB2
GABRA1
CACNA2D3
KCNA4
GABRE
GABRD
MCOLN2
TRPV2
KCNK13
CHRNA1
PKD2L1
KCNG3
CATSPER1
−0.02
−0.04
−0.06
−0.08
B Excitatory neurons
Prop-6h
Sevo-6h
Class
KCNF1
KCNC4
KCNS3
SCN1A
KCNA2
PKD2L2
SCN9A
ANO10
KCNN2
ASIC4
KCNQ5
KCNJ10
KCNH8
KCNH1
CACNA2D2
SCN7A
GABRA1
KCNE4
KCNMB1
KCNN3
KCNK1
PKD1
KCNH7
KCNT2
GLRA3
KCNJ3
CNGA3
CLIC1
KCNG3
CACNA2D3
KCNMB3
GLRA2
KCNK2
ANO7
KCNAB2
GRIK3
ANO3
KCNQ4
LRRC8B
CACNB4
−0.05
−0.1
−0.15
−0.2
−0.25
−0.3
−0.35
Prop-R2h
Sevo-R2h
Class
KCNAB2
CNGA3
GABRG1
GRIK3
KCNN2
CLCN6
KCNG3
PKD2L2
HCN1
GABRA1
KCNB2
KCNAB1
KCNH7
CLCN2
CHRNB2
KCNJ3
KCNH8
KCNQ5
KCNQ4
SCN1A
KCNA3
ASIC4
KCNS3
KCNJ10
CACNA2D2
GABRD
KCNE4
GABRA4
GLRA3
KCNJ9
KCNH1
SCN2A
GABRB1
CACNA2D3
KCND2
KCNA2
CLCN4
KCNN3
GRID2
SCN9A
−0.05
−0.1
−0.15
−0.2
−0.25
−0.3
C Interneurons
Prop-6h
Sevo-6h
Class
SCN2B
GABRB1
GABRB2
KCNAB1
GLRA2
KCND1
GRID2
CLCN5
ITPR3
ANO5
CHRNA10
KCNE5
CACNB2
SCN11A
KCNE4
KCNA4
TRPV6
PKD1L1
CATSPER4
KCNQ5
CLIC4
KCNK9
PKD2
ANO3
KCNK12
TPCN2
KCNH1
ITPR1
GRID1
CACNA1D
KCNJ8
GRIN3A
ANO7
SCN3A
ANO1
LRRC8D
KCNC4
GABRG1
LRRC8C
KCNK7
−0.04
−0.06
−0.08
−0.1
−0.12
−0.14
−0.16
Prop-R2h
Sevo-R2h
Class
GABRB1
PKD2L2
KCNJ9
ANO10
KCNK2
KCNJ13
ORAI2
KCNA2
ASIC4
KCNQ4
CLCN2
KCNAB1
KCNMB2
GABRA2
LRRC8D
SCN9A
KCNB2
KCNH7
KCNK9
GLRA3
TRPM8
KCNN2
KCND1
KCNK12
GABRG1
CACNA1D
GABRA1
CLCN5
UNC80
TRPM3
PKD1L1
ITPR1
KCND2
KCNC4
KCNN3
GABRQ
CATSPER3
CLIC2
KCNMB1
ITPR3
−0.04
−0.06
−0.08
−0.1
−0.12
−0.14
D Microglia
Prop-6h
Sevo-6h
Class
KCNAB2
SCN9A
KCNAB1
GABRB1
GABRD
LRRC8C
UNC80
KCNJ9
LRRC8D
HCN3
SCNN1D
GRIK3
KCNS3
GRID2
KCNK12
KCNK1
GABRA2
KCNT2
KCNJ3
KCNK2
GABRA4
KCNH7
KCNQ4
KCNE4
KCNMB2
KCNH8
ANO7
KCNA3
CHRNA1
CHRNB2
CACNB4
SCN7A
ANO10
KCNN3
CLCN2
CLIC4
KCNMB3
KCNC4
KCNA2
SCN1A
−0.05
−0.1
−0.15
−0.2
Prop-R2h
Sevo-R2h
Class
KCNK13
ITPR1
KCNC4
KCNAB1
CACNA2D4
KCNT2
GABRA4
CHRNA1
GABRD
KCNQ4
KCNJ9
SCN2A
CHRNB2
KCNK12
CACNA2D2
KCNMB3
CLCN2
GRIK3
ASIC4
GABRB1
CLCN6
SCN11A
KCNH7
KCNJ10
SCN1A
CACNB4
UNC80
ITPR2
CNGA3
KCNMB2
KCNE5
KCND3
KCNA2
KCNN3
PKD2
LRRC8B
SCN7A
KCNA3
LRRC8C
CACNA1E
−0.05
−0.1
−0.15
−0.2
E OPCs
Prop-6h
Sevo-6h
Class
TRPC3
SCN2A
PIEZO1
GABRD
GABRA4
GABRG2
CACNB3
GABRB1
HCN1
KCNH1
SCN7A
KCNG3
KCND1
KCNN3
KCNT1
GLRA2
CNGA3
CATSPER4
KCNJ13
TRPV6
KCNMB3
SCN11A
KCNB2
KCNC4
TRPM3
KCNE4
TRPM4
KCNMB1
ANO6
KCNJ16
SCN1A
KCND3
UNC79
KCNJ3
GABRQ
CACNG3
CACNG1
CATSPER3
ORAI1
GRIN3A
−0.05
−0.1
−0.15
−0.2
Prop-R2h
Sevo-R2h
Class
TRPC3
HVCN1
KCNA5
TPCN2
KCNQ1
CACNG1
CACNA2D3
KCNQ5
CACNB2
KCNB2
KCNA4
KCNK9
GRIN2A
TRPC4
KCNH8
KCNJ8
LRRC8C
KCNJ3
SCN11A
TRPV6
GRIN1
KCNH5
GABRA4
GABRG2
KCNC2
KCNN3
SCN7A
GRIN3A
KCNE4
KCNA1
TRPM3
KCND1
PKD2L1
CNGA3
KCNK13
KCNJ5
KCNT1
GABRE
GABRA1
KCNMB1
−0.02
−0.04
−0.06
−0.08
−0.1

Figure S11. Heatmap of cell membrane ion channel gene differences compare propofol and sevoflurane of HFC**, Related to Figure 5.**
A. Astrocytes. **B.** Excitatory neurons. **C.** Interneurons. **D.** Microglia. **E.** OPCs. Red, upregulation; blue, downregulation.

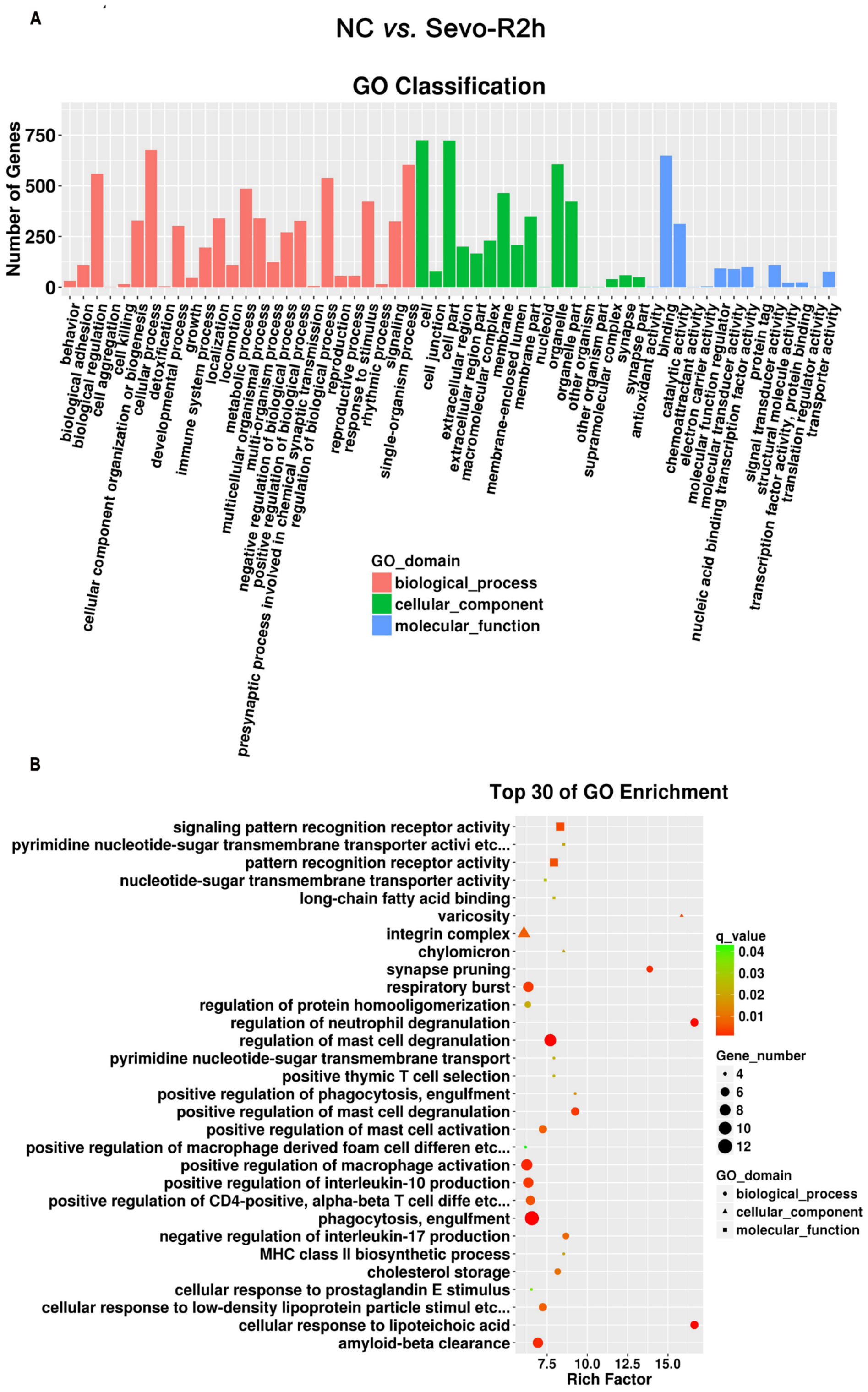

Figure S12. The RNA-seq results of NC and Sevo-R2h by GO analysis, Related to Figure 6.
A. A statistical map of GO (gene ontology) functional classification of differentially expressed genes of NC and Sevo-R2h groups. Red, biological process. Green, cellular component. Blue, molecular function.
B. The top 30 signal pathways of GO enrichment distribution points showing differentially expressed genes in NC and Sevo-R2h groups.

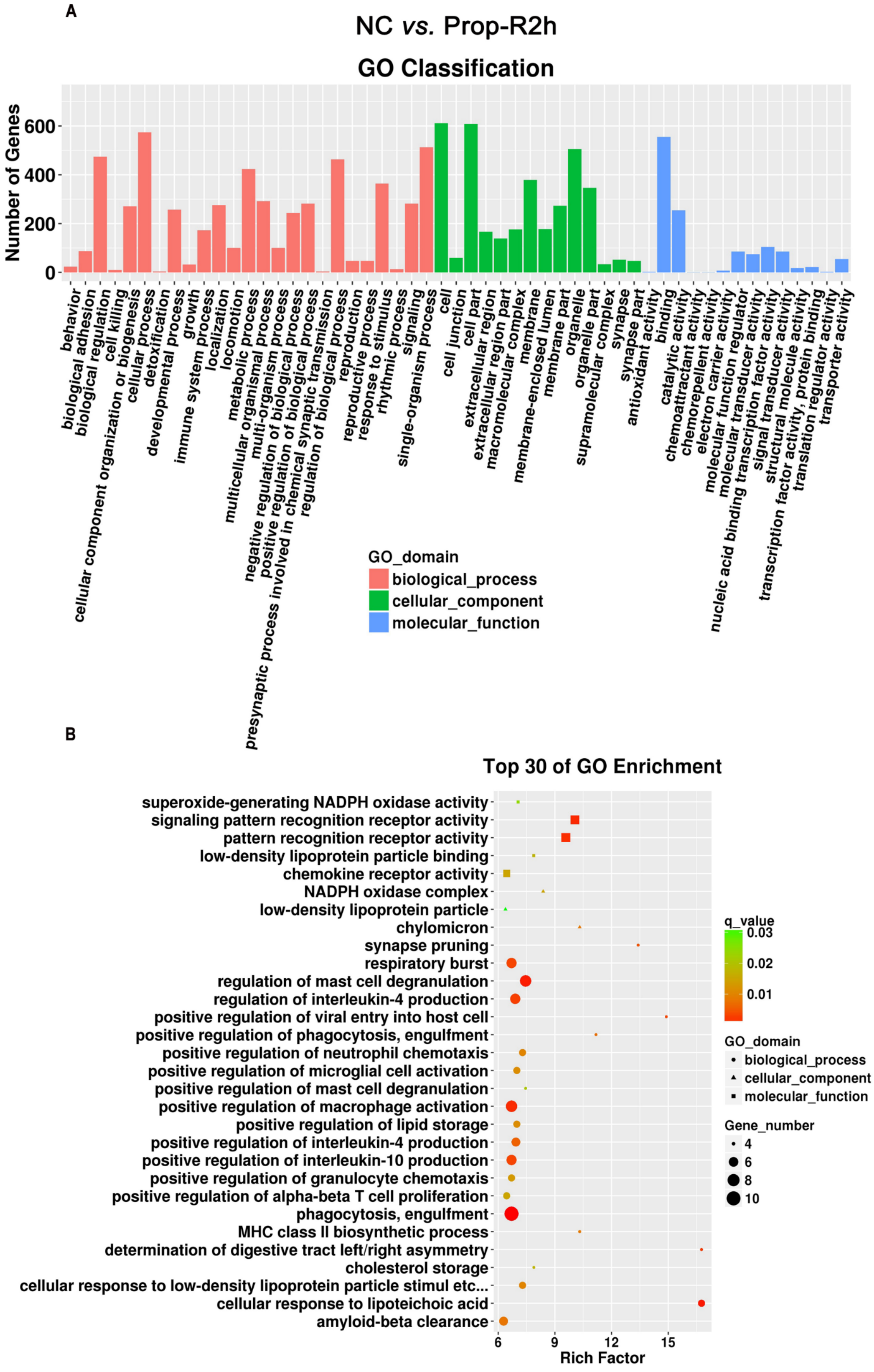

Figure S13. The RNA-seq results of NC and Prop-R2h by GO analysis, Related to Figure 6.

A. A statistical map of GO (gene ontology) functional classification of differentially expressed genes of NC and Prop-R2h groups. Red, biological process. Green, cellular component. Blue, molecular function.

B. The top 30 signal pathways of GO enrichment distribution points showing differentially expressed genes in NC and Prop-R2h groups.

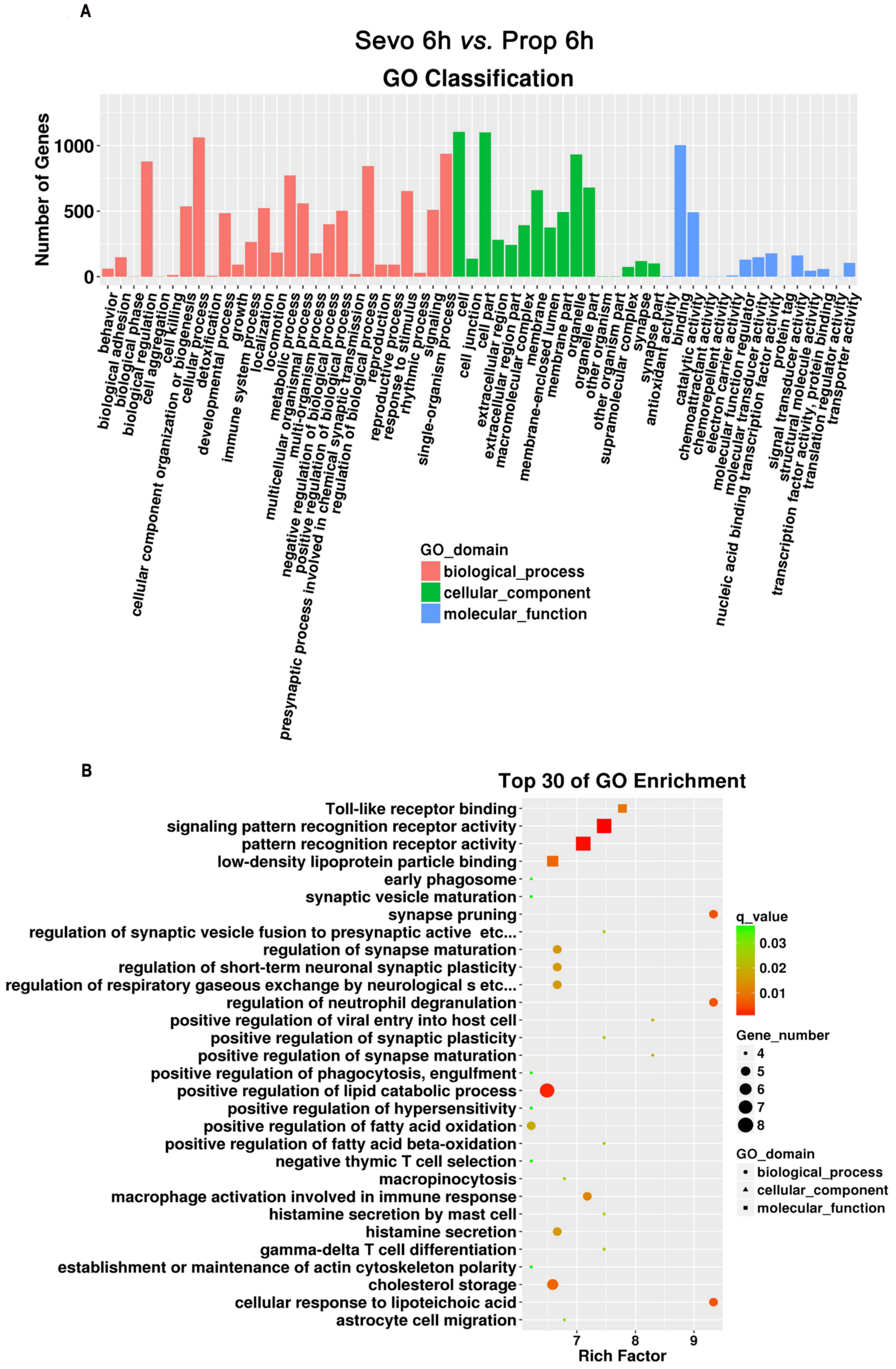

Figure S14. The RNA-seq results of Sevo 6h and Prop 6h by GO analysi, Related to Figure 6.
A. A statistical map of GO (gene ontology) functional classification of differentially expressed genes of Sevo 6h and Prop 6h. Red, biological process. Green, cellular component. Blue, molecular function.
B. The top 30 signal pathway of GO enrichment distribution points of differentially expressed genes of Sevo 6h and Prop 6h.

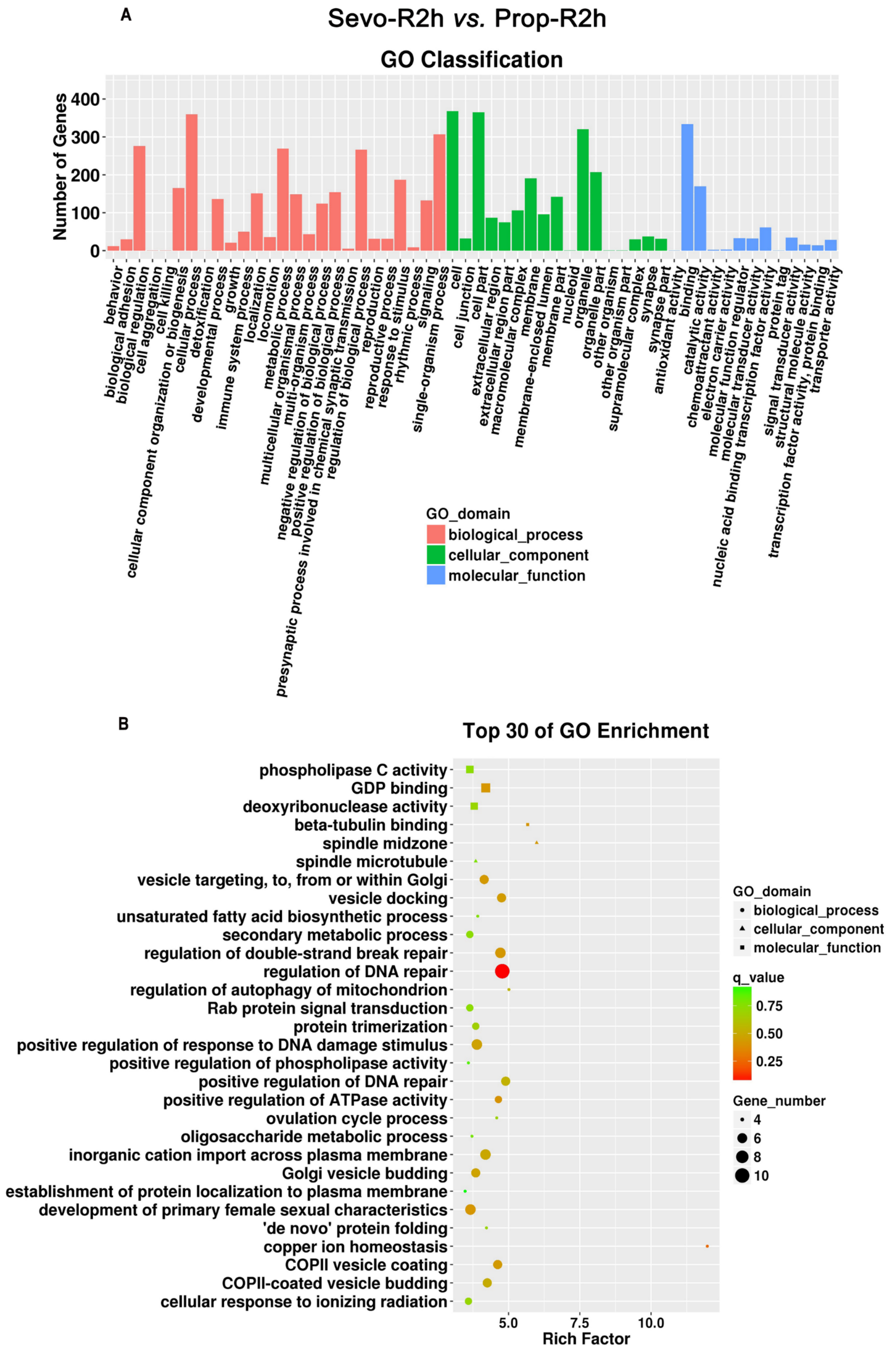

Figure S15. The RNA-seq results of Sevo-R2h and Prop-R2h by GO analysis, Related to Figure 6.

A. A statistical map of GO (gene ontology) functional classification of differentially expressed genes of Sevo-R2h and Prop-R2h. Red, biological process. Green, cellular component. Blue, molecular function.

B. The top 30 signal pathway of GO enrichment distribution points of differentially expressed genes of Sevo-R2h and Prop-R2h.

Table S1. The 10 most regulated genes by two anesthetics and their functions, Related to Figure 3.

(www.genecards.org). Brownish background part indicates the upregulated genes whilst grey part are the downregulated genes.

**Sevoflurane**	**Propofol**
ARID5A (A-T rich interaction domain 5A): involved in cell growth and tissue-specific gene expression	*CNR1* (Cannabinoid receptor 1): mediating the mood and cognition alteration effects of cannabinoids
IER2 (Immediate early response 2): involved in the regulation of neuronal differentiation	*NAV2* (Neuron navigator 2): cellular growth and migration)
FOS (Fos proto-oncogene, AP-1): regulators of cell proliferation, differentiation, and transformation	*PCLO* (Piccolo presynaptic cytomatrix protein): component of presynaptic cytoskeletal matrix to enable synaptic vesicle trafficking
PDCD10 (Programmed cell death 10): evolutionarily conserved protein associated with cell apoptosis	*SLC25A39* (Solute carrier family 25 member 39): inner mitochondrial membrane transporter
MICU2 (Mitochondrial calcium uptake 2): mitochondrial calcium uniport regulator	*IER3IP1* (Immediate early response 3 interacting protein 1): endoplasmic reticulum stress sensor that mediates cell differentiation and apoptosis
JAKMIP1 (Janus kinase and microtubule interacting protein 1): microtubule-dependent transport of the GABA-B receptor	*RTN4RL2* (Reticulon 4 receptor-like 2): cell surface receptor inhibiting axon outgrowth
RBM4B (RNA binding motif protein 4B): translational activator of circadian clock mRNA PER1	*IGFBP2* (Insulin like growth factor binding protein 2): high expression levels of this protein promote the growth of several types of tumors
PLXNA2 (Plexin A2): membrane-bound proteins that mediate repulsive effects on axon pathfinding during nervous system development	*THY1* (Thy-1 cell surface antigen): immunoglobulin involved in cell adhesion/communication of the nervous system
ATP6V1D (ATPase H+ transporting V1 subunit D): component of vacuolar ATPase for acidification of intracellular organelles	*PTP4A2* (Protein tyrosine phosphatase 4A2): cell signaling molecules that play regulatory roles in a variety of cellular processes
HIF1A (Hypoxia inducible factor 1 subunit alpha): transcriptional factor that orchestrates metabolic adaptation to hypoxia	*HIGD1A* (HIG1 hypoxia inducible domain family member 1A): the terminal component of the mitochondrial respiratory chain
